# Anandamide alters the membrane properties, halts the cell division and prevents drug efflux in multidrug resistant *Staphylococcus aureus*

**DOI:** 10.1038/s41598-021-88099-6

**Published:** 2021-04-22

**Authors:** Shreya Banerjee, Ronit Vogt Sionov, Mark Feldman, Reem Smoum, Raphael Mechoulam, Doron Steinberg

**Affiliations:** 1 Biofilm Research Laboratory, The Faculty of Dental Medicine, The Hebrew University of Jerusalem, Jerusalem, Israel; 2 The Institute for Drug Research, The Faculty of Medicine, The Hebrew University of Jerusalem, Jerusalem, Israel

**Keywords:** Microbiology, Medical research

## Abstract

Antibiotic resistance is a serious public health problem throughout the world. Overcoming methicillin and multidrug-resistant *Staphylococcus aureus* (MRSA/MDRSA) infections has become a challenge and there is an urgent need for new therapeutic approaches. We have previously demonstrated that the endocannabinoid Anandamide (AEA) can sensitize MRSA to antibiotics. Here we have studied the mechanism of action using a MDRSA clinical isolate that are sensitized by AEA to methicillin and norfloxacin. We found that AEA treatment halts the growth of both antibiotic-sensitive and antibiotic-resistant *S. aureus*. The AEA-treated bacteria become elongated and the membranes become ruffled with many protrusions. AEA treatment also leads to an increase in the percentage of bacteria having a complete septum, suggesting that the cell division is halted at this stage. The latter is supported by cell cycle analysis that shows an accumulation of bacteria in the G2/M phase after AEA treatment. We further observed that AEA causes a dose-dependent membrane depolarization that is partly relieved upon time. Nile red staining of the bacterial membranes indicates that AEA alters the membrane structures. Importantly, 4′-6-diamidino-2-phenylindole (DAPI) accumulation assay and ethidium bromide efflux (EtBr) assay unveiled that AEA leads to a dose-dependent drug accumulation by inhibiting drug efflux. In conclusion, our study demonstrates that AEA interferes with cell division, alters the membrane properties of MDRSA, and leads to increased intracellular drug retention, which can contribute to the sensitization of MDRSA to antibiotics.

## Introduction

*Staphylococcus aureus* (*S. aureus*) is a common human pathogen that is involved in a variety of infectious diseases such as: skin and soft tissue infections, endocarditis, osteomyelitis, bacteremia and lethal pneumonia. The emergence of methicillin-resistant *Staphylococcus aureus* (MRSA) and multidrug-resistant *S. aureus* (MDRSA) is a major health problem, and new strategies to combat these infections are urgent^1^. MRSA strains also possess the ability to constantly acquire additional antibiotic resistance genes, thereby creating difficult to treat superbugs^2,3^. There are several mechanisms responsible for the development of antibiotic resistance in *S. aureus*^4,5^. Among others, it may be caused by alterations of the target site resulting in reduced affinity to the antibiotics; presence of enzymes that modulate or inactivate the antibiotics; decreased uptake or enhanced efflux of the antibiotics resulting in suboptimal intracellular drug concentrations; or the bacteria are encapsulated in biofilms that reduce the bioavailability of the drugs^5–8^.

The anti-bacterial effect of penicillin and other β-lactam antibiotics is achieved by inhibiting the enzymatic activity of the transpeptidase/transglycosylase enzyme penicillin-binding protein 2 (PBP2), which is involved in the last step of cell wall synthesis^9^. Resistance to β-lactam antibiotics occurs when the bacteria express β-lactamase/penicillinase that cleaves the β-lactam ring, inactivating the drug^4^. This resistance can be overcome by the β-lactamase inhibitor clavulanic acid that is used in the combination with amoxicillin in the drug Augmentin to increase the efficiency of the penicillin^10^. Methicillin is a semisynthetic penicillin derivate that was introduced into the drug markets in the late 1950s since it is not degraded by penicillinase^5^. However, resistance to methicillin rapidly developed that led to its discontinuation. The resistance to methicillin is due to the acquisition of a non-native gene (*mecA*) that encodes for the transpeptidase PBP2a showing low affinity for methicillin and other β-lactam antibiotics, thereby enabling cell wall synthesis in the presence of β-lactams^4,5,9^. The *mecA* gene is carried on a mobile genetic element termed staphylococcal chromosomal cassette (*SCCmec*) that also express the two genes *mecR1* and *mecI* involved in the regulation of *mecA*^5^. Only the 5th generation of cephalosporin β-lactams (e.g., ceftaroline and ceftobiprole) show a sufficiently high affinity for PBP2a^5^. But also here, bacteria emerge that have acquired resistance to these antibiotics through mutations in *pbp2*, *pbp4* and *gdpP*^11^. Another cause of β-lactam resistance is the expression of *mecC* that encodes for a PBP2a/2′ variant^12^.

Norfloxacin is a fluoroquinolone that stabilizes DNA-DNA gyrase and DNA-topoisomerase IV complexes, thereby blocking the movement of the DNA-replication fork, resulting in the inhibition of DNA replication^13^. In *S. aureus*, topoisomerase IV is the primary quinolone target^13^. Resistance to fluoroquinolones can appear when the binding sites of these two enzymes are mutated. Another frequent resistance mechanism is the overexpression of the NorA, NorB and NorC efflux pumps^14^.

Various efflux pumps are involved in the extrusion of toxic substrates, including antibiotics and waste products, which is driven by an energy-dependent process^8,15^. The multidrug efflux systems are classified into five families according to their energy requirements and structures^15^. The major facilitator superfamily (MFS), the small multidrug resistance (SMR) family, the multidrug and toxic compound extrusion (MATE) family, and the resistance-nodulation-cell division (RND) superfamily use the proton motive force to drive the extrusion of their substrates by an anti-port H^+^:drug mechanism^15,16^. The MATE transporters can also use Na^+^ membrane gradient instead of H^+^^15,16^. On the other hand, the transporters of the adenosine-triphosphate (ATP)-binding cassette (ABC) superfamily use ATP to drive the extrusion of their substrates^15,16^. Usually, each kind of efflux pump shows specificity toward certain groups of antibiotics and/or other antimicrobial compounds^8,15,16^. For instance, NorA-C, MepA and MdeA can pump out fluoroquinolones such as ciprofloxacin and norfloxacin as well as quaternary ammonium compounds (QACs), while the efflux pumps SepA and QacA/B can extrude QACs and biguanidines (e.g., chlorhexidine)^8,15^. Many of the efflux pumps can also extrude dyes such as ethidium bromide (EtBr), Hoechst 33342 and rhodamine which fluoresce and thereby enable the measurement of efflux pump activity^17–19^. Several drugs and natural products have been shown to sensitize resistant bacteria to antibiotics by either inhibiting the activity of efflux pumps or downregulating their expression^16,20,21^. Another mechanism for drug sensitization is the disassembly of the membrane microdomains of MRSA by the cholesterol-lowering drugs of the statin family, which ultimately results in inactive monomeric PBP2a^22^.

There are accumulating data supporting a role of efflux pumps in biofilm formation^23–25^. The efflux pumps are involved in the excretion of extracellular matrix molecules and quorum sensing molecules that coordinate biofilm formation of various bacterial species^25^. MexAB-OprM of *Pseudomonas aeuroginosa* and AdeFGH of *Acinetobacter baumannii* are examples of efflux pumps that play important roles in biofilm formation^25^. The multidrug-resistance ABC transporter AbcA in *S. aureus* is involved in the secretion of phenol-soluble modulins (PSMs)^26^, that comprise the structural scaffold of *S. aureus* biofilms^27^. AbcA also confers resistance to β-lactam antibiotics such as nafcillin and methicillin^28^. The expression of the *norB*, *norC* and *mdeA* efflux pump genes were found to be upregulated in *S. aureus* during biofilm growth^29^. Another hint at a relationship between efflux pumps and biofilm formation comes from the study showing that the pleiotropic regulator MgrA in *S. aureus* negatively regulates the expression of the efflux pumps *norB* and *norC*^28^ and represses biofilm formation^30^. This is further supported by the observation that the efflux pump inhibitors (EPIs) chlorpromazine, thioridazine, carbonyl cyanide m-chlorophenylhydrazone (CCCP) and phenyl-arginine- β-naphthylamide (PAβN) inhibited biofilm formation of *S. aureus*^23,24^. EPIs might also overcome the inherent antibiotic resistance of the bacteria embedded in the biofilms^24,31,32^.

We have previously shown that the endocannabinoid anandamide (Arachidonoyl ethanolamine; AEA) may act on *S. aureus* by reducing its ability to produce biofilms^33^, and more importantly, it was shown to sensitize methicillin-resistant *S. aureus* (MRSA) to various antibiotics including ampicillin, methicillin, tetracycline and gentamicin^34^. Recently, we also observed that AEA prevented yeast-hyphal transition of *Candida albicans* and prevented the adherence of filamentous *C. albicans* to cervical epithelial cells^35^.

AEA is an endogenous lipid signaling molecule derived from arachidonic acid that was originally detected as a retrograde compound in the brain that is secreted by the post-synaptic neuron and acts on pre-synaptic neurons to regulate the neurotransmitter levels in the synapsis^36–38^. In mammals, it acts as an agonist on the cannabinoid receptors CB1 and CB2, and can activate the vanilloid receptor TRPV1^39^. AEA has been shown to have anti-anxiety and anti-depressant activities^40,41^. In addition, it exerts immunosuppressive properties^42–45^. Intraperitoneal administration of AEA could attenuate the development of ulcerative colitis in mice^45^. In the present research, we have looked for the mechanisms involved in the AEA sensitization of MDRSA to antibiotics.

## Results

### The susceptibility of different *S. aureus* strains to methicillin and other antibiotics

**Figure 1:**
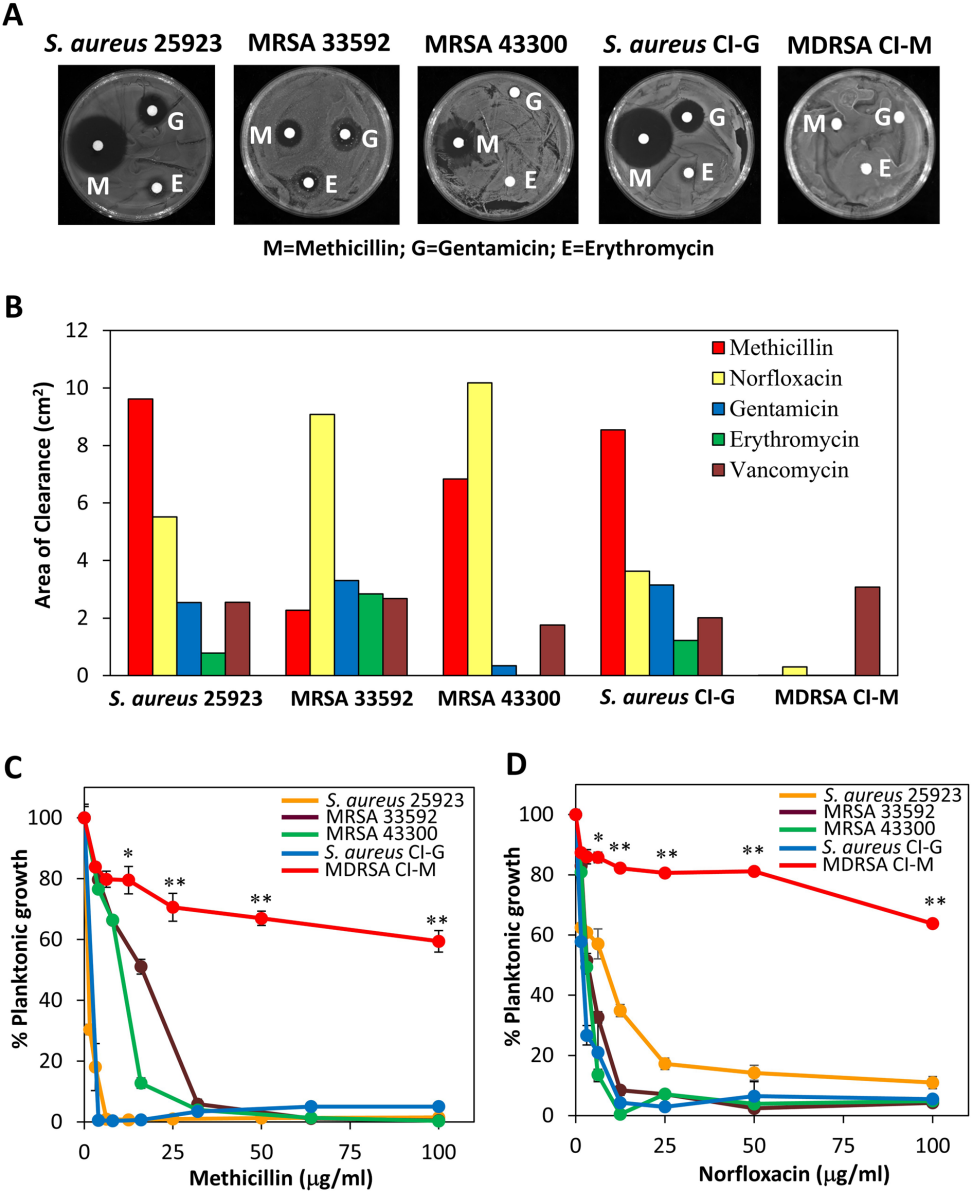
Sensitivity of different *S. aureus* strains to antibiotics. (**A**) Disc diffusion assay using circled Whatman paper containing 50 μg of the indicated antibiotics. *M* Methicillin, *G* Gentamicin, *E* Erythromycin. (**B**) A summary of the disc diffusion assay. (**C**,**D**) The susceptibility of planktonic growing bacteria to increasing doses of methicillin (**C**) or norfloxacin (**D**). The OD_595nm_ of the samples was measured after a 24 h incubation. *p < 0.05; ** p < 0.001.

Initially we searched for a clinical isolate of *S. aureus* that exhibited multidrug resistance. We compared the susceptibility of two clinical isolates (CI-G and CI-M) to five different antibiotics with that of three well-defined ATCC strains (*S. aureus* MSSA 25923, MRSA 33592 and MRSA 43300) using the disk diffusion assay (Fig. 1A,B) and the broth microdilution method (Fig. 1C,D and Table 1). Whereas CI-G was highly sensitive to methicillin, norfloxacin and gentamicin at a comparable level to that of the antibiotic-sensitive *S. aureus* strain 25923, CI-M showed high resistance to these antibiotics. Methicillin and norfloxacin reduced the planktonic growth of CI-M to 30–40% at the highest concentrations tested after a 24 h incubation (Fig. 1C,D). Furthermore, CI-M showed high resistance to erythromycin, while being susceptible to vancomycin (Fig. 1B and Table 1). Since CI-M shows high resistance to four different classes of antibiotics with a MIC above 100 µg/ml, we have considered it as a multidrug-resistant *S. aureus* (MDRSA) according to the definition of ECDC/CDC^46^.

**Table 1:** The MIC of MDRSA CI-M to various antibiotics and AEA in comparison to other *S. aureus* strains.

	*S. aureus* 25923	MRSA 33592	MRSA 43300	*S. aureus *CI-G	MDRSA CI-M
Methicillin	6.25 µg/ml	32 µg/ml	32 µg/ml	4 µg/ml	> 500 µg/ml
Norfloxacin	50 µg/ml	12.5 µg/ml	12.5 µg/ml	12.5 µg/ml	> 100 µg/ml
Gentamicin	12.5 µg/ml	100 µg/ml	> 100 µg/ml	100 µg/ml	> 100 µg/ml
Erythromycin	> 100 µg/ml	> 100 µg/ml	> 100 µg/ml	> 100 µg/ml	> 100 µg/ml
Vancomycin	1.56 µg/ml	3.1 µg/ml	3.1 µg/ml	> 100 µg/ml	3.1 µg/ml
AEA	> 100 µg/ml	100 µg/ml	> 100 µg/ml	> 100 µg/ml	> 500 µg/ml

*MIC* Minimum inhibitory concentration that leads to invisible bacterial growth.

### Anandamide (AEA) sensitizes MDRSA CI-M to methicillin and norfloxacin

**Figure 2:**
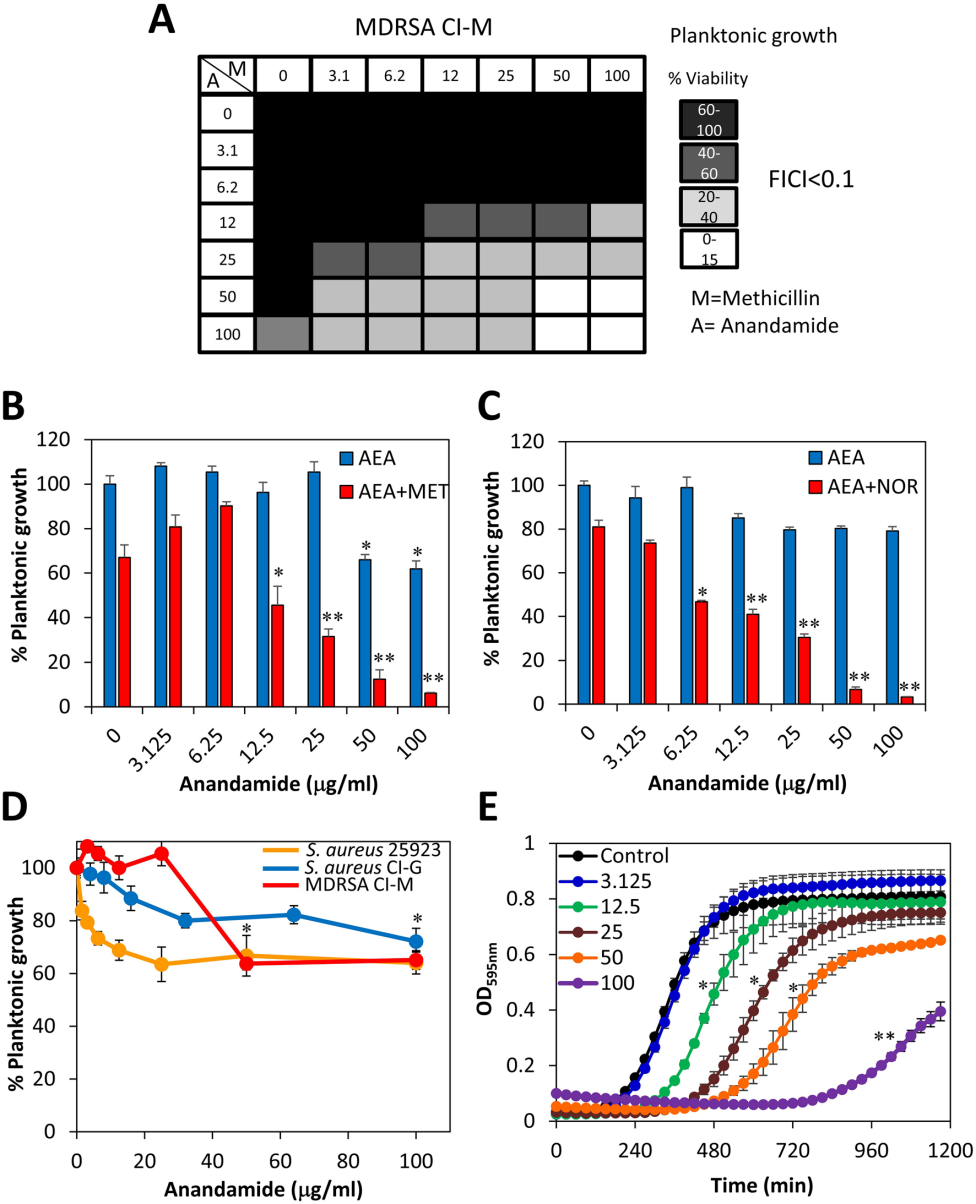
Anandamide (AEA) sensitizes MDRSA CI-M to methicillin and norfloxacin. (**A**) The survival of planktonic growing MDRSA CI-M after a 24 h incubation in a checkerboard assay of AEA with methicillin. *A* Anandamide; *M* Methicillin. (**B**) The survival of planktonic growing MDRSA CI-M in the absence or presence of 50 μg/ml methicillin (MET) and increasing concentrations of AEA. (**C**) The survival of planktonic growing MDRSA CI-M in the absence or presence of 50 μg/ml norfloxacin (NOR) and increasing concentrations of AEA. (**D**) The survival of planktonic growing MDRSA CI-M in the presence of increasing concentrations of AEA alone. (**E**) A kinetic study of the planktonic growth of MDRSA CI-M in the presence of increasing concentrations of AEA. *p < 0.05; **p < 0.01.

Our previous study showed that AEA could sensitize MRSA 33592, MRSA 43300 and a MRSA clinical isolate to methicillin, ampicillin, gentamicin and tetracycline^34^. Here, we confirmed that AEA could also sensitize MDRSA CI-M to methicillin (Fig. 2A,B) and norfloxacin (Fig. 2C) using a checkerboard assay where the bacteria were exposed to increasing concentrations of the antibiotics and AEA in different combinations. The FICI value was lower than 0.1 when the AEA concentration was equal to and above 25 μg/ml, indicative for a synergistic effect. Also, by calculating the data according to the method of Weinstein et al.^47^, the percentage killing observed in the presence of both components was higher than the additive effect of each, again indicating a synergistic effect. When studying the effect of AEA on the survival of the bacteria in an endpoint planktonic growth assay after a 24 h incubation, there was a modest dose-dependent reduction in the survival that didn't exceed 40% for both MDRSA CI-M and the antibiotic-sensitive *S. aureus* strains 25923 and CI-G (Fig. 2D). The MIC of AEA was found to be above 500 µg/ml for MDRSA CI-M. There was no significant difference in the growth of the different strains in the presence of AEA, suggesting that this effect of AEA is independent of the antibiotic sensitivity status of the bacteria. A kinetic planktonic growth of MDRSA CI-M in the presence of increasing concentrations of AEA, showed a dose-dependent lag in the log-phase growth curve (Fig. 2E), suggesting there is an initial inhibition of cell growth that was overcome at later time points. While the log-phase growth of control cells initiated at around 3 h, the log-phase growth of 12.5, 25, 50 and 100 μg/ml AEA-treated bacteria commenced after 5.5, 7.0, 8.5 and 13.5 h, respectively (Fig. 2E). A similar halt in cell division by AEA was observed in the *S. aureus* 25923 antibiotic-sensitive strain (data not shown), indicating that this is caused by a direct effect of AEA on the bacterial growth.

**Figure 3:**
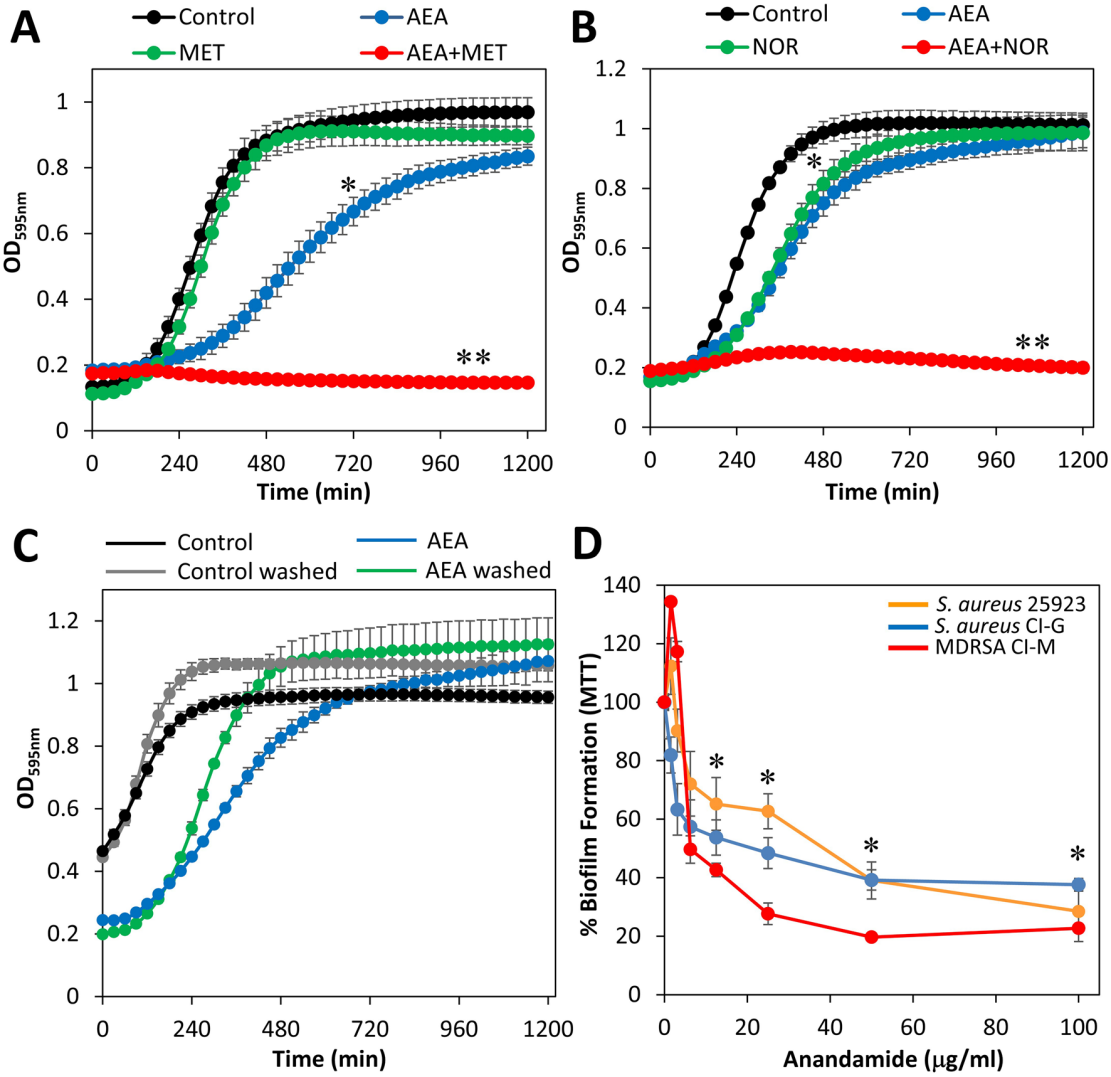
(**A**) A kinetic study of the planktonic growth of MDRSA CI-M in the absence or presence of 50 μg/ml AEA and/or 50 μg/ml methicillin for 20 h. (**B**) A kinetic study of the planktonic growth of MDRSA CI-M in the absence or presence of 50 μg/ml AEA and/or 50 μg/ml norfloxacin for 20 h. (**C**) MDRSA CI-M at an OD of 0.1 was incubated in the presence of 50 μg/ml AEA for 4 h, then the AEA medium from half of the culture was replaced with fresh growth medium (washed), while the other half of the culture remained with AEA. The same procedure was done for control bacteria. This was followed by a kinetic study of the planktonic growth for 20 h. (**D**) The effect of AEA on the biofilm formation by the three *S. aureus* strains MSSA 25923, MSSA CI-G and MDRSA CI-M. After a 24 h incubation of the bacteria with increasing concentration of AEA, the metabolic activity of the formed biofilms was measured using the MTT assay. *p < 0.05; **p < 0.01.

Next we performed a kinetic growth curve study for the combined effect of AEA and the antibiotics methicillin or norfloxacin (Fig. 3A,B). Methicillin at 50 µg/ml did not affect the planktonic growth of MDRSA CI-M (Fig. 3A), while norfloxacin at 50 µg/ml showed a similar delay in the log growth phase as 50 µg/ml AEA (Fig. 3B). Only when both AEA and either of the two antibiotics were present, there was a prominent growth inhibition (Fig. 3A,B), which conforms to the data presented in Fig. 2B,C showing AEA-mediated sensitization of MDRSA CI-M to the antibiotics.

We also analyzed whether a pretreatment with AEA is sufficient to cause prolonged bacteriostatic effect. To this end, MDRSA CI-M was pretreated with 50 µg/ml AEA for 4 h, after which AEA was removed and the bacteria resuspended in fresh growth medium. This was followed by a kinetic study of the planktonic growth (Fig. 3C). The growth of the AEA-pretreated bacteria was compared to that of continuous AEA treatment and of control bacteria that was also given refreshed growth medium. The AEA-pretreated bacteria showed a significant growth arrest for around 2.5–3 h, and then entered the log growth phase (Fig. 3C). In the log growth phase, the division rate of the AEA-pretreated bacteria was higher than those being continuously exposed to AEA (Fig. 3C), suggesting that the bacteriostatic effect is reversible and the growth inhibited bacteria can recover when AEA is removed. It should be noted that the replacement with fresh medium causes a growth advantage as seen by more profound growth of the washed control bacteria in comparison to control bacteria grown in the same medium throughout the experiment (Fig. 3C).

### The anti-biofilm activity of AEA in the absence or presence of antibiotics

**Figure 4:**
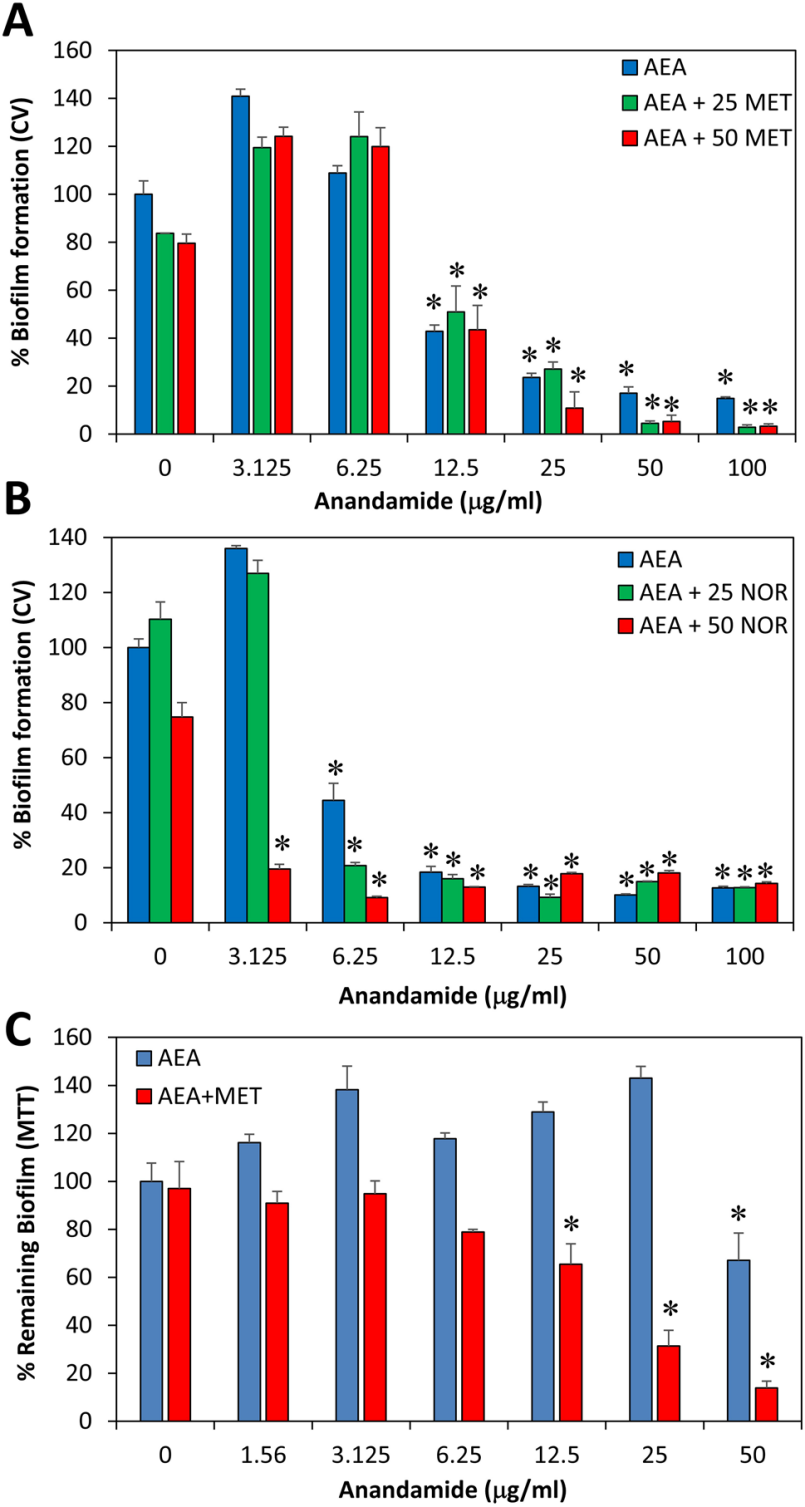
The effect of combined treatment of MDRSA CI-M with AEA and antibiotics on biofilm formation. (**A**) Biofilm formation after a 24 h incubation of MDRSA CI-M in the absence or presence of 25 or 50 μg/ml methicillin (MET) and increasing concentrations of AEA as measured by crystal violet staining. (**B**) Biofilm formation after a 24 h incubation of MDRSA CI-M in the absence or presence of 25 or 50 μg/ml norfloxacin (NOR) and increasing concentrations of AEA as measured by crystal violet staining. (**C**) MDRSA CI-M was allowed to form biofilms for 24 h. The biofilms were then exposed to 50 μg/ml methicillin (MET) and increasing concentrations of AEA for another 24 h and the metabolic activity of the remaining biofilms was measured using the MTT assay. *p < 0.05.

Previous studies in our laboratory have shown potent anti-biofilm activity of AEA on biofilm formation and preformed biofilms of three MRSA strains^33^. We also observed that some combinations of AEA and antibiotics had synergistic anti-biofilm effect which was dependent on the MRSA strain and the kind of antibiotics used^34^. Here we show that AEA has a profound anti-biofilm activity against MDRSA CI-M, as well as the *S. aureus* strains 25923 and CI-G (Fig. 3D), indicating that the anti-biofilm effect of AEA is independent of the antibiotic-susceptibility status of the bacteria. Next we studied the effect of the combined treatment of AEA with methicillin or norfloxacin on biofilm formation by MDRSA CI-M (Fig. 4A,B). Methicillin did not significantly enhance the anti-biofilm effect of AEA (Fig. 4A), while at the low AEA concentrations, norfloxacin exerted a synergistic anti-biofilm effect (Fig. 4B). Importantly, when preformed biofilms of MDRSA CI-M were treated with AEA in combination with methicillin, a higher eradication of the preformed biofilms was observed than either drug alone (Fig. 4C).

### AEA increases the cell size and alters the membrane structure of MDRSA CI-M

**Figure 5:**
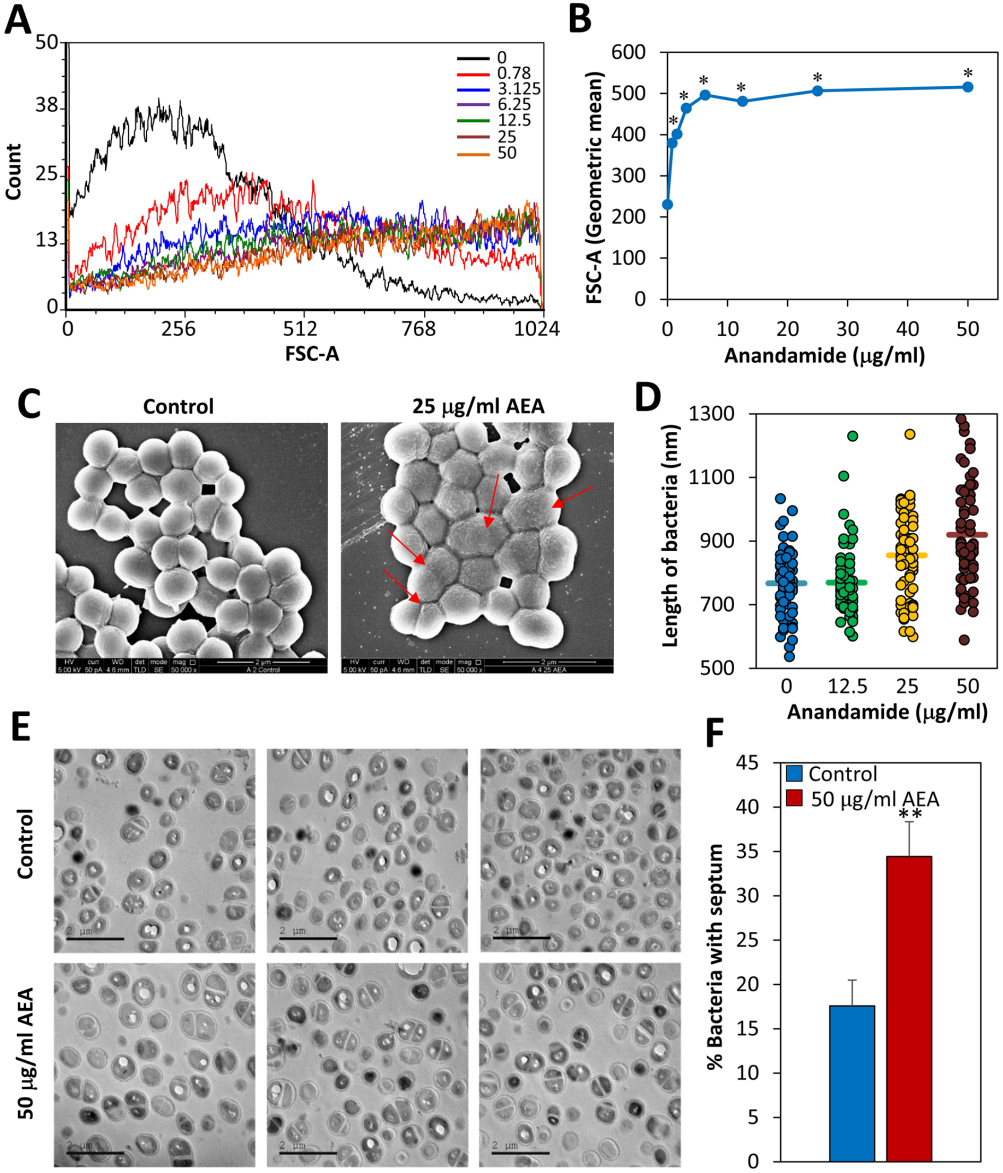
AEA changes the structure of the bacteria and increases the number of bacteria with complete septum. (**A**) Forward scatter on flow cytometry of bacteria treated with various concentrations of AEA for 2 h. (**B**) The graph shows the mean geometric FSC of data presented in (**A**). (**C**) SEM images of control bacteria or bacteria treated with 25 μg/ml AEA for 2 h. The red arrows point to bacteria that are larger and seem to be in division, but did not yet separate. The AEA-treated bacteria show ruffled membranes, which is in contrary to the control bacteria with smooth membranes. (**D**) The length of the bacteria after a 2 h treatment with AEA compared to control bacteria. n = 65–70 for each treatment group measured from 4–5 independent images. The horizontal lines present the average size. The y axis is set within the range of the cell sizes. (**E**) TEM images of control and AEA-treated bacteria for 2 h. (**F**) The graph presents the percentage of bacteria showing complete septum. 840–890 bacteria were counted in 6 different images of each sample. **p < 0.002.

When running flow cytometry of AEA-treated bacteria after a 2 h incubation, we observed a striking shift in the forward scatter (FSC) to the right even at the low concentration of 0.78 μg/ml (Fig. 5A,B). The side scatter was similar (data not shown). The shift in FSC suggests there is an increased cell size in response to AEA treatment. To explore this possibility, control and AEA-treated bacteria after a 2 h incubation were visualized by high-resolution scanning electron microscopy (HR-SEM) (Fig. 5C and Suppl. Figure 1). Indeed, we observed that the bacteria become larger with several bacteria showing signs of septum without daughter cell separation (Fig. 5C). In addition, the membrane become ruffled with many protrusions (Fig. 5C). This is in contrast to control bacteria that show smooth membranes and dominant circular morphology (Fig. 5C). The cell length of the control bacteria varied between 530–1044 micron with an average of 765 micron (Fig. 5D), while the length of the bacteria treated with 50 μg/ml AEA for 2 h ranged from 590–1340 micron with an average length of 920 micron (Fig. 5D).

### AEA leads to the accumulation of bacteria in the late stage of cell division

**Figure 6:**
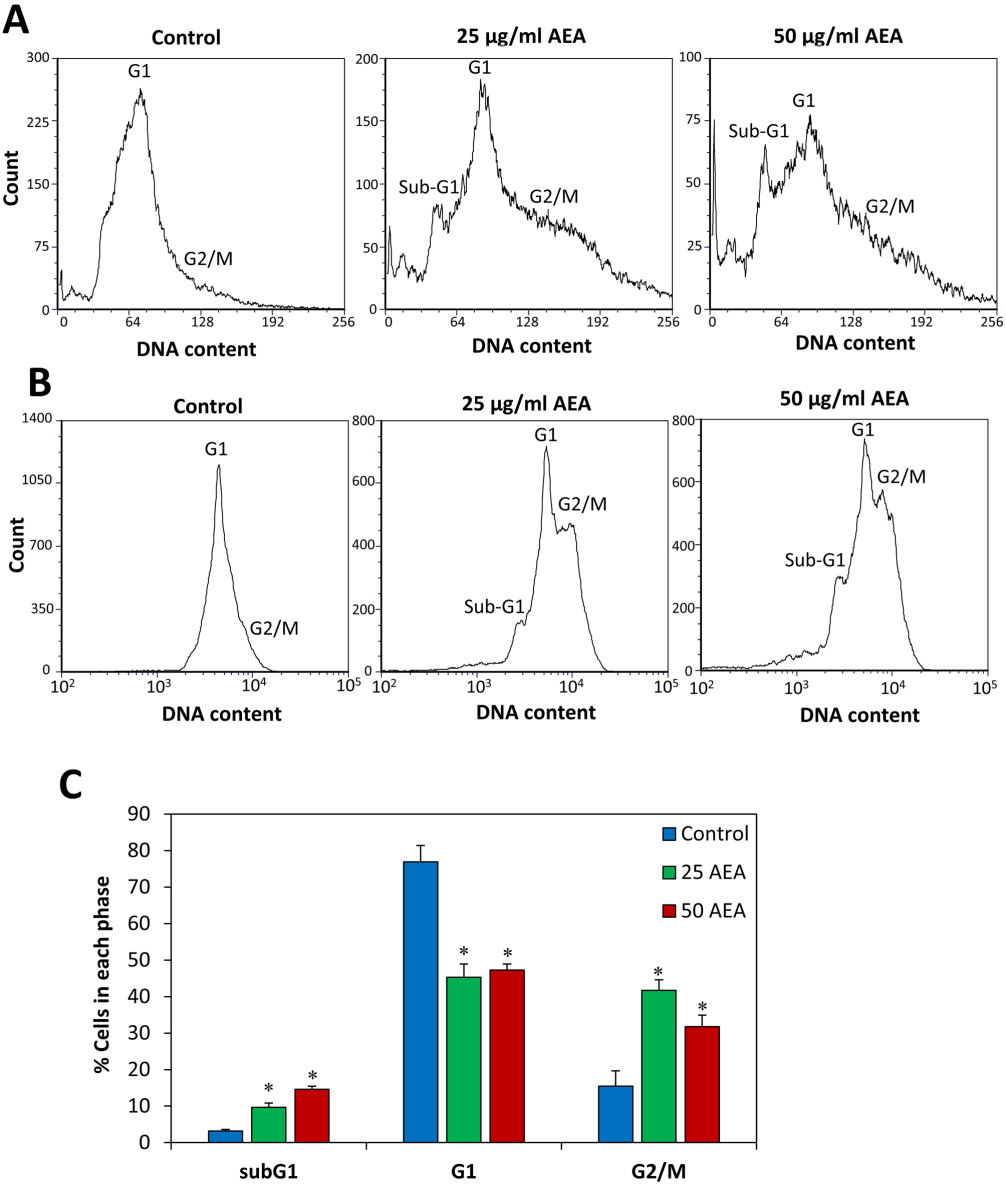
AEA leads to the accumulation of bacteria in the G2/M phase of the cell cycle. (**A**,**B**) MDRSA CI-M was incubated in the absence or presence of the indicated concentrations of AEA for 2 h, and then fixed with methanol and stained with DAPI. (**A**) The cell cycle read in linear scale. (**B**) The cell cycle read in logarithmic scale. (**C**) The percentage of bacteria in Sub-G1, G1 and G2 as measured from 4 different samples. *p < 0.05.

To further understand the effect of AEA on cell division, we examined the control and AEA-treated bacteria after a 2 h incubation by a transmission electron microscope (TEM) (Fig. 5E and Suppl. Figure 2). The images show higher percentage of bacteria with a complete septum in the AEA-treated bacteria (34.4% ± 3.9%) in comparison to control bacteria (17.6% ± 2.9%) (Fig. 5E,F). These data suggest that AEA interferes with the late stage of cell division. In order to substantiate this observation, we performed cell cycle analysis of MDRSA CI-M that has been treated with 50 µg/ml AEA for 2 h and compared it to untreated control bacteria. The cell cycle analysis was performed either in linear scale (Fig. 6A) or logarithmic scale (Fig. 6B). In both cases, most of the control bacteria appeared in the G1 cell cycle phase with a small tail to the right representing the G2/M phase (12–20%; Fig. 6A,B). This goes in line with studies showing short retention time in the G2/M phase in *S. aureus*^48^. Interestingly, AEA treatment led to a profound accumulation of the bacteria in the G2/M phase (30–45%; Fig. 6A,C), with concomitant reduction in bacteria in the G1 phase. There was also an increase in the sub-G1 cell population in the presence of 25–50 µg/ml AEA (9–15%; Fig. 6A,C), suggesting that AEA has induced cell death in this subpopulation of the bacterial culture. The increase in the percentage of bacteria retained in the G2/M phase accords with the TEM data suggesting an inhibition of the cell division at the late stage of cell division. Intriguingly, the percentage of bacteria in the G2/M phase is similar to the percentage of bacteria with complete septum as observed in the TEM images.

### AEA didn’t inhibit β-lactamase activity

One common resistance mechanism towards penicillin is the expression of β-lactamase that inactivates β-lactam antibiotics^15^. Although methicillin resists degradation by this enzyme^5^, it was prompting to study whether AEA could affect the activity of β-lactamase. To this end, control and AEA-treated bacteria after a 30 min incubation were exposed to the β-lactamase chromogenic substrate nitrocefin, and the hydrolysis of nitrocefin was monitored each minute for 60 min. The β-lactamase activity was not interrupted by any of the concentrations of AEA studied (Suppl. Figure 3), suggesting that the sensitization to penicillin such as ampicillin^34^ is caused by a different mechanism.

### Effect of AEA on gene expression

**Figure 7:**
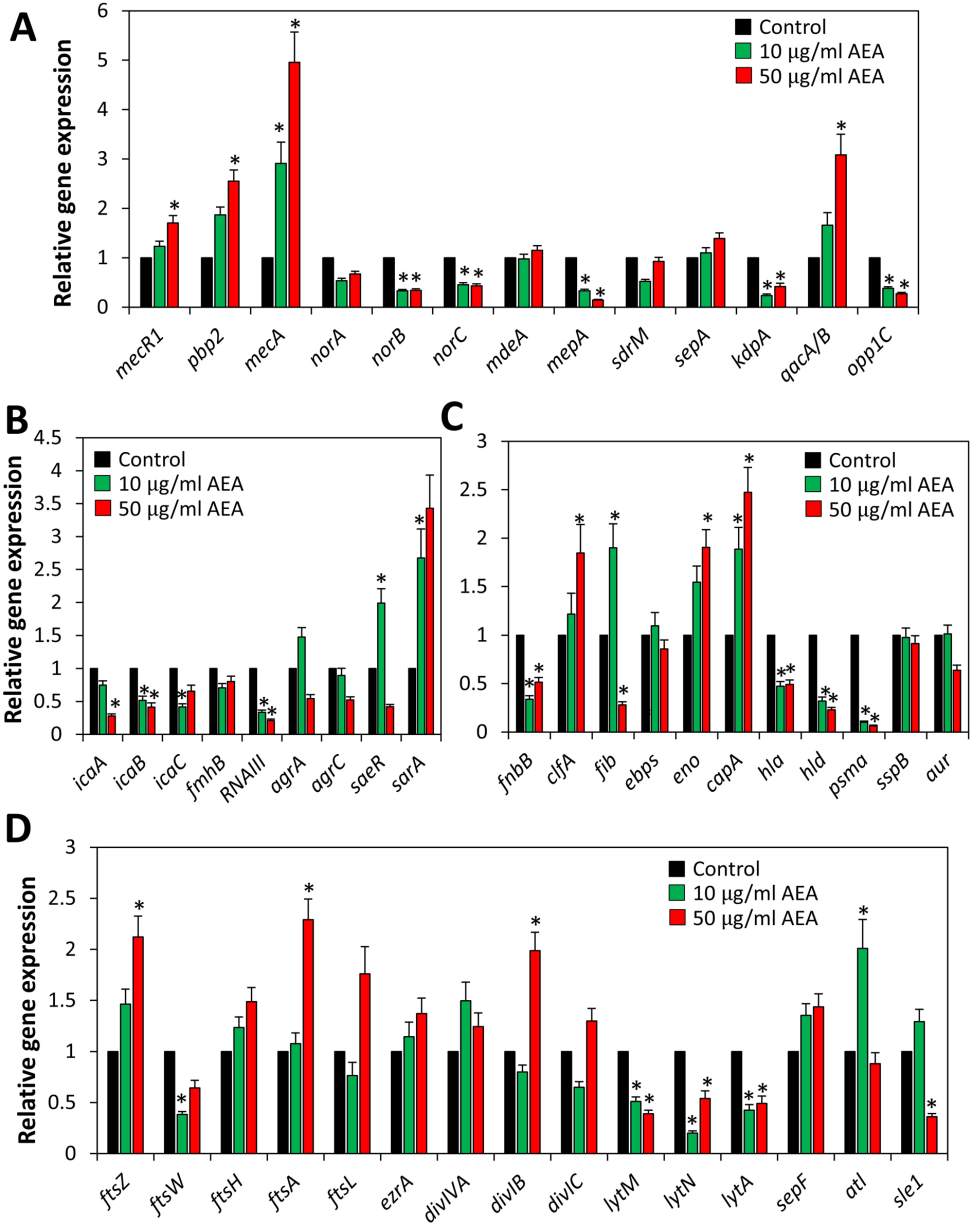
Real-time qPCR gene analysis of MDRSA CI-M that has been treated with AEA for 4 h in comparison to control bacteria. The graphs present the average fold-change as calculated against the seven house-keeping genes *gyrB*, *glyA*, *gmk*, *proC*, *recF*, *rho* and *RNAII*. (**A**) Genes related to antibiotic resistance including efflux pumps. (**B**) Regulatory and biofilm-related genes. (**C**) Virulence genes. (**D**) Genes related to cell division. *p < 0.05.

We further explored whether the sensitization to antibiotics could be due to altered expression of genes related to antibiotic resistance. We surprisingly observed that AEA after a 4 h incubation upregulated the expression of both *pbp2* and *mecA* (*pbp2a*) that are involved in cell wall synthesis (Fig. 7A). Also the gene *qacA/B* encoding for the efflux pump involved in the extrusion of quaternary ammonium compounds and biguanidines^15^ was upregulated by AEA (Fig. 7A). On the contrary, some other efflux pump genes were downregulated with a particular significant effect on the multidrug export protein *mepA*, the potassium transporter *kdpA* and the oligopeptide transporter *opp1C* (Fig. 7A). Among the biofilm-related and quorum sensing genes studied, we observed a strong reduction in expression of the regulatory RNAIII, while the staphylococcal accessory regulator A (*sarA*) was upregulated by AEA (Fig. 7B). Next, we analyzed the effect of AEA on various virulence genes and found that *hld* encoding for delta-hemolysin and the amphipathic α-helical phenol-soluble modulin *psmα* were strongly downregulated (Fig. 7C). In light of our finding showing that AEA affects the bacterial growth, we also examined the expression of cell division-associated genes. AEA caused a two-fold increase of the *ftsZ*, *ftsA* and *divIB* that form part of the divisome^49^, while the expression of the hydrolase enzymes *lytM*, *lytN* and *lytA* important for daughter cell separation^50^ were significantly repressed (Fig. 7D). The latter may provide an explanation for the AEA-mediated interference of the late stage of cell division.

### AEA reduces the membrane potential

**Figure 8:**
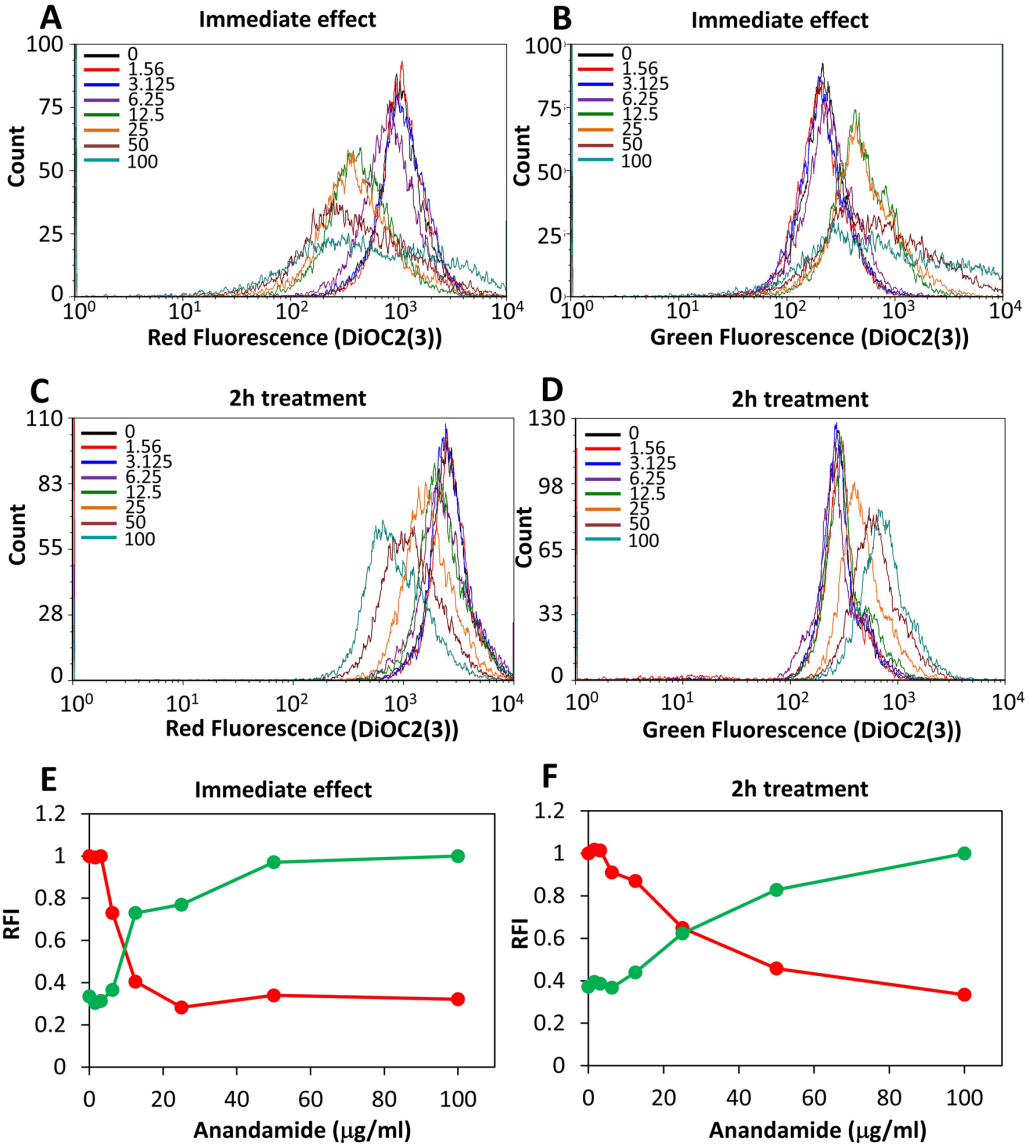
AEA causes an immediate drop in membrane potential that was partially relieved after 2 h. (**A**–**D**) The membrane potential was measured using the DiOC2(3) dye on flow cytometry immediately after adding AEA (**A**,**B**) and after 2 h treatment with AEA (**C**,**D**). Red fluorescence is an indication for the strength of the membrane potential. (**E**,**F**) are summaries of the relative fluorescence intensity (RFI) of the red (red lines) and green (green lines) fluorescence for the immediate AEA effect (**E**) and after 2 h treatment with AEA (**F**).

The membrane potential of MDRSA CI-M was measured immediately after adding AEA or after a 2 h pretreatment with AEA using the DiOC2(3) reagent. AEA caused an immediate dose-dependent decrease in the red fluorescence intensity with a simultaneous increase in the green fluorescence intensity, indicating an immediate reduction in membrane potential by AEA (Fig. 8A,B,E). After a 2 h incubation of the bacteria with AEA, the membrane potential was still lower than that of the control bacteria (Fig. 8C,D,F), but higher than at time 0. These data indicate that the membrane potential is partly recovered with time even in the presence of AEA.

### AEA leads to altered membrane structure and causes DAPI accumulation in MDRSA CI-M

**Figure 9:**
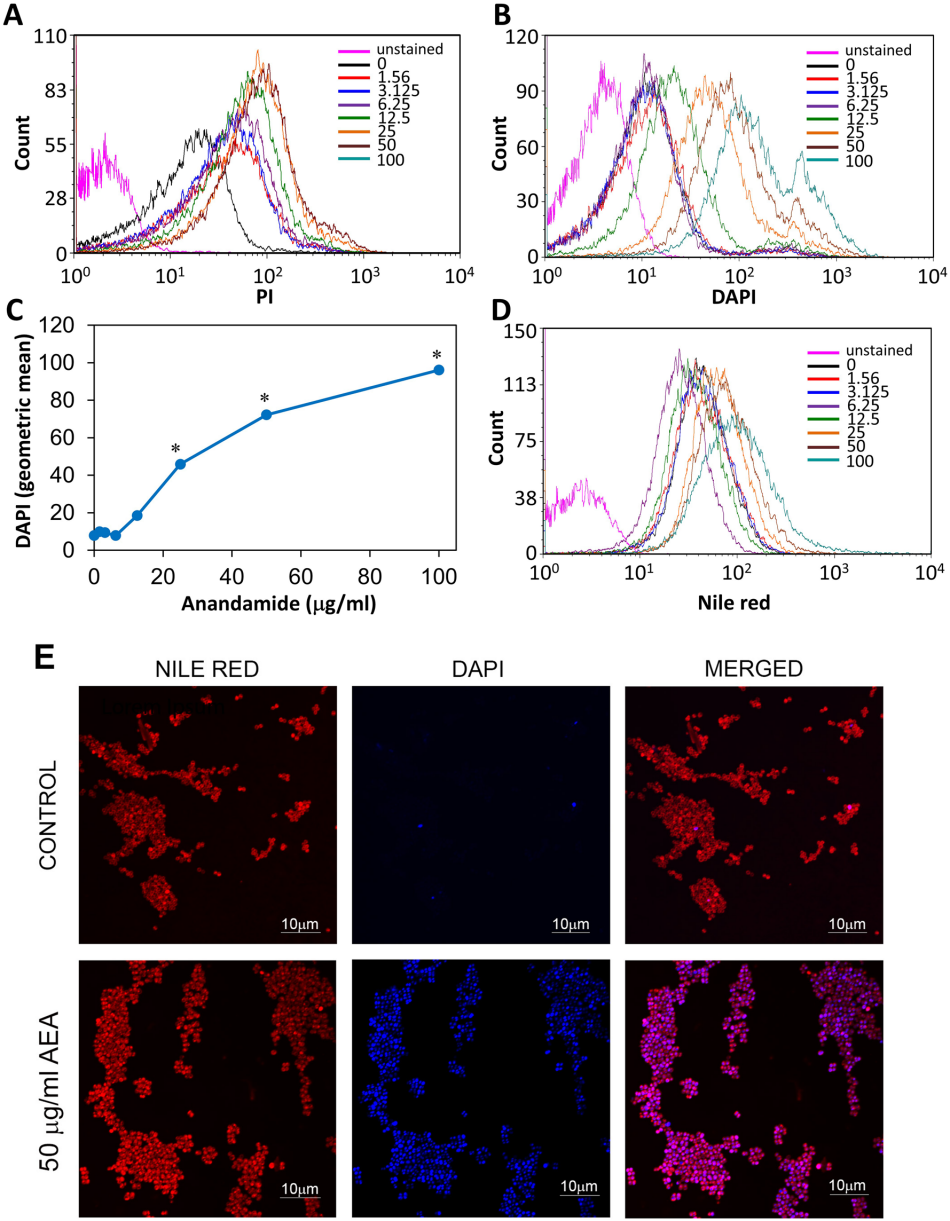
AEA leads to a dose-dependent accumulation of DAPI. (**A**) Propidium iodide (PI) uptake of MDRSA CI-M treated with increasing concentrations of AEA for 2 h as determined by flow cytometry. (**B**) DAPI uptake of MDRSA CI-M treated with increasing concentrations of AEA for 2 h as determined by flow cytometry. (**C**) A summary of the geometric mean fluorescence intensity of DAPI accumulation in the AEA-treated bacteria presented in B. (**D**) The fluorescence intensity of nile red membrane staining of control and AEA-treated bacteria as determined by flow cytometry. (**E**) Spinning disk microscopy of control and 2 h AEA-treated MDRSA CI-M that were stained with nile red (red fluorescence) and DAPI (blue fluorescence). *p < 0.05.

Next we studied whether AEA affected the membrane permeability in MDRSA CI-M using the propidium iodide (PI) probe which only enters the cells if the membrane is perforated. Flow cytometry data shows that there is a slight increase in PI fluorescence intensity of the AEA-treated bacteria after a 2 h incubation in comparison to the control bacteria (Fig. 9A), but the effect was not dose-dependent. The control bacteria showed basal PI fluorescence when compared to unstained bacteria (Fig. 9A). However, when the AEA-treated bacteria after a 2 h incubation were stained with DAPI, there was a dose-dependent increase in DAPI fluorescence intensity at doses equal to and above 12.5 μg/ml (Fig. 9B,C), suggesting that DAPI is accumulating in the AEA-treated bacteria. Concomitant nile red staining of the bacterial membranes showed increased nile red fluorescence intensity at doses equal to and higher than 25 μg/ml AEA in comparison to control bacteria (Fig. 9D), which corresponds with the data that AEA-treated bacteria become longer in size (Fig. 5C,D). Scanning disk microscopy of control and AEA-treated MDRSA CI-M that has been stained with nile red and DAPI shows different membrane structure in the AEA-treated bacteria (Fig. 9E). Whereas the control bacteria show classical circled red fluorescence at the bacteria periphery, the AEA-treated bacteria show dotted areas suggesting for unequal membrane distribution (Fig. 9E). This finding suggests that AEA affects the membrane properties. When examining the DAPI staining, we can clearly see that the DAPI staining is intracellular and accumulated within the AEA-treated bacteria (Fig. 9E). Scattered DAPI-positive cells were seen in the control bacteria (Fig. 9E). The accumulation of DAPI is a sign that AEA prevents its efflux.

### AEA inhibits the efflux of EtBr

**Figure 10:**
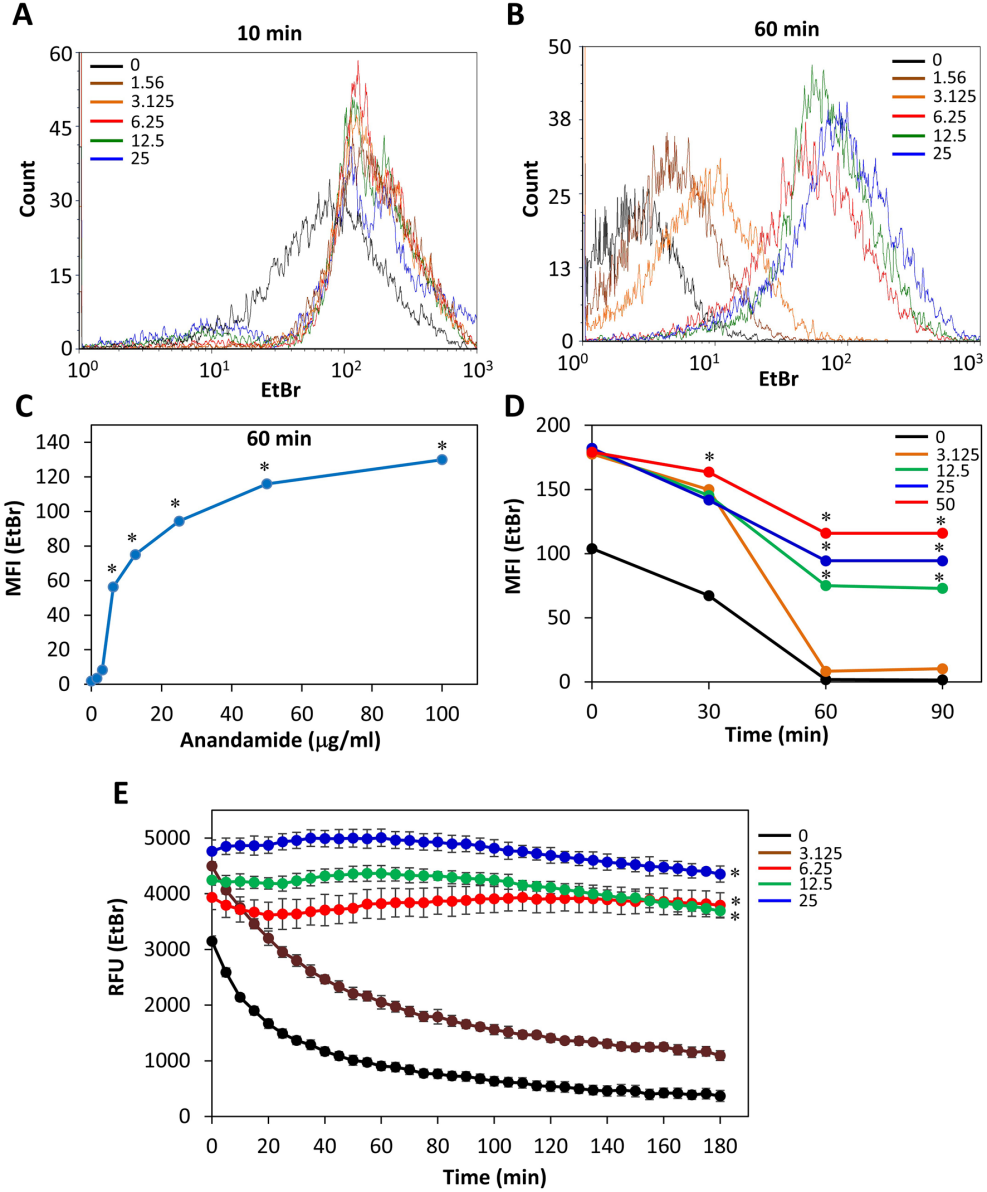
AEA prevents the efflux of ethidium bromide (EtBr) from MDRSA CI-M. (**A**,**B**) Flow cytometry of EtBr retained in MDRSA CI-M 10 min (**A**) and 60 min (**B**) after treating the bacteria with different concentrations of AEA. (**C**) The summary of the geometric mean fluorescence intensity (MFI) of samples shown in (**B**). (**D**) The MFI of the various treatments in a time course study using flow cytometry. (**E**) A time course study measuring the fluorescence intensity (RFU) of EtBr using the Tecan plate reader. *p < 0.05.

A possibility for the sensitization of AEA to antibiotics could be inhibition of the efflux pumps. Most of the efflux pumps of *S. aureus* can use EtBr as a substrate^17^. Since EtBr only fluoresces when bound to DNA, we can distinguish between the intracellular and extracellular EtBr. The MDRSA CI-M was loaded with EtBr and then exposed to various concentrations of AEA. Flow cytometry was performed to monitor the intracellular EtBr content at various time intervals (Fig. 10A–D). After 10 min both control and AEA-treated bacteria showed high EtBr content, although the AEA treated bacteria showed a higher fluorescence intensity than the control bacteria (Fig. 10A). After 30 min, most of the EtBr has been expelled from the control bacteria, while still high EtBr content was observed in the samples treated with doses equal to and higher than 12.5 μg/ml AEA (Fig. 10B,C). The EtBr remained at high levels in the AEA-treated bacteria even after 120 min (data not shown). We also undertook a kinetic study to measure the fluorescence over time in control and AEA-treated bacteria using a fluorescent plate reader (Fig. 10E). Also this assay clearly demonstrates that AEA prevents the efflux of EtBr from the cells, which is an indication that AEA inhibits the efflux pumps.

### AEA increases intracellular norfloxacin level and reduces cell wall synthesis

**Figure 11:**
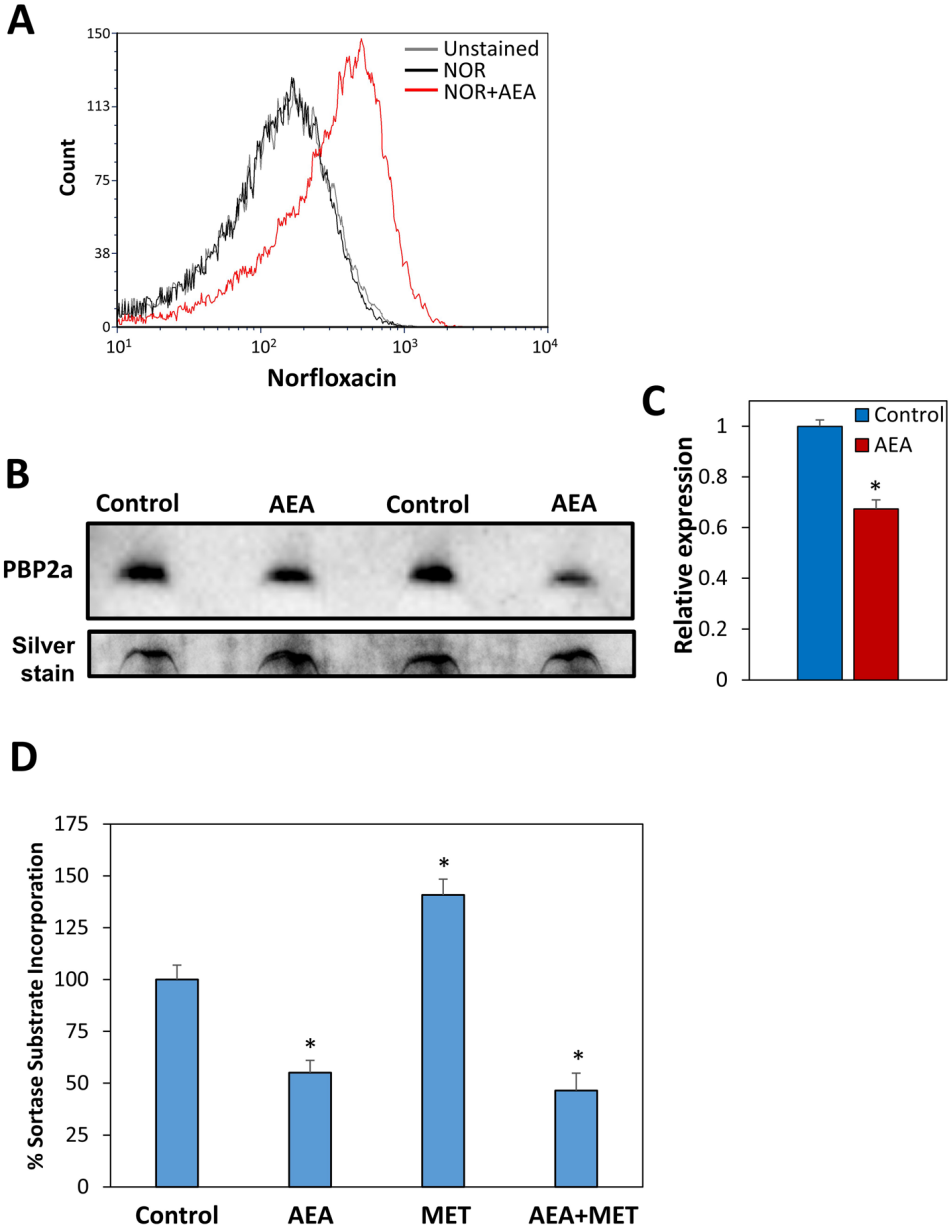
(**A**) AEA increases the intracellular level of norfloxacin. MDRSA CI-M was incubated with 25 µg/ml Norfloxacin (NOR) in the absence or presence of 25 µg/ml AEA for 2 h at 37 °C. Then the norfloxacin was removed, and the amount of norfloxacin retained in the bacteria was measured after 3 h on flow cytometry. (**B**) PBP2a expression levels (upper panel) in crude membrane extracts of MDRSA CI-M treated with 50 µg/ml AEA for 2 h in comparison to control. Two different samples of control and AEA-treated bacteria are shown. Lower panel: Silver staining of the same samples in the polyacrylamide gel as loading control. (**C**) The relative PBP2a expression level in AEA-treated versus control MDRSA CI-M as measured from 4 different samples using the Photoshop software on the PBP2a panel shown in (**B**). (**D**) Inhibition of cell wall synthesis by AEA. MDRSA CI-M was incubated with 50 μg/ml AEA and/or 50 μg/ml methicillin (MET) for 2 h, and then incubated with Sortase Substrate I, FRET, for 2.5 h. At the end of incubation, the fluorescence intensity (Ex/Em = 340/490) of the washed bacteria was measured in a Tecan plate reader. * p < 0.05.

Since we observed that AEA inhibits drug efflux, it was querying to study the effect of AEA on the intracellular level of norfloxacin. For this purpose, MDRSA CI-M was loaded with norfloxacin in the absence or presence of AEA for 2 h, and 3 h after removing norfloxacin from the growth medium, the remaining intracellular level of norfloxacin was measured by flow cytometry based on its property of being a fluorescent drug. Indeed, we observed that AEA resulted in the retention of norfloxacin within the bacteria in comparison to untreated control bacteria (Fig. 11A), which might provide an explanation for the drug sensitization.

Since the real-time PCR analysis showed an increase in *mecA* (*pbp2a*) gene expression in response to AEA (Fig. 7A), we analyzed the PBP2a protein level after AEA treatment. For this purpose, we prepared crude membrane extracts of control and AEA-treated bacteria after a 2 h incubation, and the PBP2a level was measured by Western blotting. In contrast to the gene expression studies, the PBP2a protein level was found to be 30% lower in the AEA-treated bacteria than in the control samples (Fig. 11B,C; Suppl. Figure 4). This suggests that the observed increase in *mecA* (*pbp2a*) gene expression could be a compensation mechanism to the downregulation of its protein level.

It was also of interest to study the effect of AEA on cell wall synthesis. To this end, MDRSA CI-M was exposed to 50 µg/ml AEA in the absence or presence of 50 µg/ml methicillin for 2 h followed by a 2.5 h incubation with Sortase substrate I. At the end of incubation, the fluorescence intensity of the bacteria was measured. This study shows that AEA reduces the cell wall synthesis, and also prevents the methicillin-induced upregulation of cell wall synthesis (Fig. 11D). Thus, despite of the AEA-mediated upregulation of the *mecA* (*pbp2a*) gene transcripts, the cell wall synthesis was inhibited by AEA.

## Discussion

Biofilm formation is a major cause for treatment failure of persistent bacterial infections. *Staphylococcus* species are frequently involved in the formation of these biofilms. These bacteria often develop multidrug resistance (MDR)^51^. Multidrug resistance has become a major challenge in treating bacterial infections, and new therapeutic strategies are required to combat these diseases. One strategy to overcome this obstacle is to use drugs that can sensitize the bacteria to the antibiotics. We have recently observed that the endocannabinoid anandamide (AEA) not only reduces biofilm formation of MRSA^33^, but also sensitizes MRSA to methicillin, ampicillin, gentamicin and tetracycline^34^. The aim of the present study was to understand the mechanisms by which AEA sensitizes the antibiotic-resistant bacteria to antibiotics. For this purpose, we looked for a clinical isolate of multidrug resistant *S. aureus* that could be used in this study. First, we compared the antibiotic sensitivity of two clinical isolates with three ATCC defined *S. aureus* strains, and found that the clinical isolate CI-M showed high resistance to four different classes of antibiotics, and therefore can be classified as MDRSA^46^. The other clinical isolate CI-G was highly sensitive to all of the five antibiotics tested, comparable to the ATCC 25923 antibiotic-sensitive strain. It was important to confirm that AEA could also sensitize the MDRSA CI-M strain to antibiotics similar to the previously observed sensitization of MRSA to antibiotics^34^. Indeed, we observed that the AEA sensitizes MDRSA to both methicillin and norfloxacin, indicating that AEA is a broad-acting antibiotic sensitizing compound. We also confirmed that AEA exerted anti-biofilm activities toward MDRSA CI-M, and the combined treatment of preformed biofilms with AEA and methicillin, could eradicate these biofilms.

Kinetic growth studies showed that AEA in a dose dependent manner delayed the initiation of bacterial log growth phase, suggesting that AEA impedes cell division. This delay in bacterial growth was similar in both antibiotic-sensitive and antibiotic-resistant *S. aureus*, indicating that this is due to a direct effect of AEA on the bacteria, independent of the antibiotic resistance mechanisms. Strikingly, when we studied the AEA-treated bacteria on flow cytometry, we observed a higher forward scatter of the AEA-treated bacteria in comparison to control bacteria, suggesting that the bacteria has become larger in size. Indeed, high-resolution scanning electron microscopy (HR-SEM) confirmed that the bacteria become longer at the average after the short period of 2 h incubation with AEA. Moreover, the AEA-treated bacteria appeared with an irregular membrane that had many small protrusions, suggesting that AEA affects the membranes properties. The membrane effect of AEA is further emphasized by nile red staining that showed dotted membrane structures in the AEA-treated bacteria in contrast to the clear uniform peripheral membrane staining of the control bacteria. Since AEA is a lipophilic compound known to integrate into the membrane where it can interact with different membrane-associated proteins^52^, it is not surprising that AEA profoundly alters the membrane structures. This can also explain why AEA causes an immediate depolarization of the membrane potential. AEA-treated bacteria showed only a slightly higher propidium iodide incorporation in comparison to control bacteria, suggesting that it does not lead to a significant increase in membrane permeability.

The HR-SEM images also showed signs that the bacteria attempted to divide, but had difficulties separating from each other, which can explain their larger sizes. This impact on cell division was confirmed by transmission electron microscopy demonstrating twice the number of bacteria with complete septum in the AEA-treated samples in comparison to the control samples. The accumulation of bacteria with complete septum in the AEA-treated samples suggests that AEA interferes with the late phase of cell division where daughter cell separation occurs. This notion is substantiated by cell cycle analysis demonstrating that AEA treatment leads to an accumulation of bacteria in the G2/M phase. When studying the expression of genes associated with cell division, we observed significant upregulation of *ftsZ*, *ftsA*, and *divIB* that are important for proper function of the divisome^49^, while the autolytic hydrolases *lytM*, *lytN* and *lytA* important for daughter cell separation^50^ were downregulated by AEA. The increase in the cell division genes might be a compensatory mechanisms to the bacteriostatic effect of AEA, and might facilitate the resumption of cell growth at later time points. The reduced expression of autolytic hydrolases might, at least in part, explain the arrest in cell division at the stage of daughter cell separation.

In addition to the effect of AEA on the membrane properties and cell division, we raised the question whether AEA affected the expression of genes related to antibiotic resistance. Surprisingly we observed that AEA significantly upregulated the expression of penicillin binding protein-2 (*pbp2*) involved in cell wall synthesis and *mecA* (*pbp2a*), a pbp2 analog that shows low affinity to β-lactams and, in such, can proceed cell wall synthesis even in the presence of β-lactam antibiotics. AEA also led to the upregulation of *qacA/B* involved in the efflux of quaternary ammonium compounds and biguanidines^15^. On the other hand, AEA downregulated the expression of some other efflux pumps such as multidrug export protein *mepA*, the potassium transporter *kdpA* and the oligopeptide transporter *opp1C*. While the downregulation of these efflux pumps can contribute to drug retention, the upregulation of *pbp2* and *mecA* (*pbp2a*) can’t explain the AEA sensitization of MDRSA to methicillin. We therefore analyzed the protein level of PBP2a after a 2 h incubation with AEA. In contrast to the increased level of *pbp2a* transcripts, the PBP2a protein level was reduced by AEA. Thus, there was no correlation between the *pbp2a* transcript and its protein level. The reduced PBP2a level might contribute to the reduced cell wall synthesis observed in the presence of AEA.

Strikingly, we observed that AEA led to a dose-dependent accumulation of DAPI within the bacteria. DAPI has a similar structure to Hoecht 33342, which is a well-characterized efflux pump substrate^53^, meaning that the retention of DAPI within the AEA-treated bacteria is likely due to an inhibitory activity on efflux pumps. We further confirmed the efflux pump inhibitory effect of AEA by using the EtBr efflux pump assay^17^. The MDRSA were preloaded with EtBr and then treated with AEA, and the amount of EtBr retained within the bacteria was measured in kinetic studies. The EtBr was expelled from the control MDRSA, while being retained within the MDRSA in a dose- dependent manner after treatment with AEA. We also observed that AEA leads to increased intracellular level of norfloxacin in comparison to control bacteria, which seems to be related to the AEA-mediated inhibition of efflux pumps. The prolonged increase in the intracellular norfloxacin level by AEA, might contribute to the AEA sensitization of MDRSA to this antibiotic. It is likely that also other antibiotic drugs are positively affected by the efflux pump inhibition. This could be the case for tetracycline that is known to be effluxed by NorB, TetK and Tet38 in *S. aureus*^28^. Since efflux pumps are involved in secreting essential components of the biofilm matrix, it is likely that efflux pump inhibition by AEA contributes to its profound anti-biofilm activity. Such a mechanism has recently been suggested by Zimmermann et al.^31^ to be involved in the anti-biofilm activity of the NorA efflux inhibitor nilotinib.

In conclusion, we have observed in this study that AEA can sensitizes MDRSA to antibiotics such as methicillin and norfloxacin. AEA acts at several levels. First, it impedes cell growth by interfering with the late stage of cell division. Second, it affects the membrane structures that become rough and ruffled with many small protrusions. Third, AEA leads to an instant membrane depolarization. Fourth, AEA inhibits efflux pump activities resulting in increased drug concentration within the bacteria.

## Material and methods

### Materials

Anandamide (AEA) was synthesized as described before^54^. AEA (> 97.0% purity) was also purchased from Sigma-Aldrich. Other compounds ordered from Sigma include: carbonyl cyanide m-chlorophenylhydrazone (CCCP), ethidium bromide (EtBr), propidium iodide (PI), 4′,6-diamidine-2′-phenylindole dihydrochloride (DAPI), gentamicin, vancomycin, norfloxacin and erythromycin. Methicillin was purchased from MedChemExpress. AEA, gentamicin, vancomycin, erythromycin and methicillin were prepared as 10 mg/ml stock solution in ethanol. Respective ethanol dilutions were used as control. A 10 mg/ml norfloxacin solution was prepared in 50 mM acetic acid. Control samples were treated with respective concentrations of acetic acid. EtBr, PI and DAPI were dissolved in DDW.

### Microbial strains and growth conditions

The microbial strains used in this study were MSSA *S. aureus* ATCC 25923, MRSA ATCC 33592, MRSA ATCC 43300, *S. aureus* clinical isolate G (CI-G), and MDRSA clinical isolate M (CI-M). The latter was defined as MDRSA as it is insensitive to methicillin, norfloxacin, gentamicin and erythromycin, and it expresses multiple efflux pumps as determined by real-time PCR. The day before experiment, frozen bacterial stock were inoculated in tryptic soy broth (TSB) (Acumedia, Neogen) at a ratio of 1:100 and incubated at 37 °C overnight until an OD_600nm_ of 1.8–2.0 was reached. At the following day the OD_600nm_ was measured using Ultraspec10 (Amersham Biosciences) and the bacteria were centrifuged and resuspended in TSB supplemented with 1% D-glucose (TSBG) to an OD_600nm_ of 0.05–0.5 depending on the assay. For all experiments, the same bacterial culture was used for both control and treated samples that were incubated in parallel for the same time periods with identical initial OD and under the same incubation conditions. Control samples got the same dilutions of ethanol as the treated samples.

### Minimum inhibitory concentration (MIC) assay

MIC was determined by a micro-broth dilution method in 96-flat bottomed clear tissue culture-grade microplate (Corning)^34^. 200 μl of bacteria in TSBG at an OD_600nm_ of 0.05 were incubated in a twofold increase of the test compound concentration ranging from 1 to 100–500 μg/ml for 24 h at 37 °C. At the end of incubation, the OD_595nm_ was measured in a Tecan Infinite M200 microplate reader using the iControl software. Blank samples of the test compounds at the different concentrations without bacteria were included to correct for basal OD values. For kinetic studies, the OD_595nm_ was measured every 30 min for a period of 20 h in the Tecan M200 microplate reader at 37 °C. The MIC of each compound was defined as the lowest concentration that inhibited visible growth of the bacteria (ISO 17025 standard).

### Checkerboard assay

The synergistic antibacterial effect of AEA with methicillin or norfloxacin was analyzed by checkerboard assay essential as described^34^. Briefly, 100 μl of bacteria at an OD_600nm_ of 0.1 were added to each well in 96-flat bottom clear tissue culture-grade microplates. Fifty μl of two-fold dilutions of AEA were added in the vertical directions, while 50 μl of two-fold dilutions of methicillin or norfloxacin were added in the horizontal directions. All combinations were tested in triplicates and in the concentration ranging from 1 to 256 μg/ml for 24 h at 37 °C. At the end of incubation, the OD_595nm_ was measured in a Tecan M200 microplate reader. A synergistic effect was calculated by two different methods. One is the fractional inhibitory concentration (FIC) method^55^. Here the ∑FICs were calculated by the following formula: ∑FIC = FIC_A_ + FIC_B_, where FIC_A_ is the MIC of drug A in the combination/MIC of drug A alone, and FIC_B_ is the MIC of drug B in the combination/MIC of drug alone. The drug combination is considered synergistic when the ∑FIC index is equal or below 0.5. The other method is to use the null hypothesis for an additive effect, and if the observed effect is stronger than the additive effect, it is considered synergistic^47^.

### Disk diffusion antibiotic sensitivity test

The disk diffusion antibiotic sensitivity test^56^ was performed to determine the antibiotic sensitivity pattern of the different bacterial strains. Fifty microgram in 5 μl of the different antibiotics were applied to round Whatman 1 mm paper discs with a diameter of 5 mm. These discs were applied on TBS agar plates on which 100 μl of an overnight bacterial culture has been spread. The plates were incubated for 24 h at 37 °C, and the zone of inhibition was measured.

### Biofilm formation and quantification of the biofilm mass

An overnight culture of MDRSA CI-M was resuspended in TSBG to an OD of 0.05 and incubated with different concentrations of the test compounds in 200 μl TSBG in 96 flat-bottom well microplates (Corning). After a 24 h incubation, the biofilms formed at the bottom of the wells were washed with PBS and the biofilm mass was either determined by crystal violet staining or by the MTT metabolic assay^33,34,57^. To determine the effect of the test compounds on preformed biofilms, the biofilms were allowed to form in 96 flat-bottom well microplates for 24 h as described above. After washing the biofilms twice with 200 µl sterile PBS, the biofilms were incubated with different concentrations of the test compounds in 200 µl TSBG and incubated at 37 °C for another 24 h. At the end of the incubation, the biofilms were washed twice with 200 µl PBS and the metabolic activity in the remaining biofilms was measured by the MTT assay.

### Crystal violet staining

To quantify the biofilm biomass, the washed biofilms were stained with 200 μl of a 0.1% crystal violet solution (1:4 dilution in DDW of the Gram's crystal violet solution, Merck) for 20 min at room temperature^57^. The stained biofilms were washed twice with DDW, and after drying the wells, the stain was dissolved in 200 μl of a 33% acetic acid solution, and quantified by reading the OD at 595 nm in the M200 Tecan microplate reader. Different dilutions of the test compounds in the absence of bacteria were used to measure background signals. The percentage biofilm formation was calculated by dividing the OD of treated samples on OD of control samples, multiplied with 100%.

### MTT staining

The metabolic activity of biofilms was examined using the MTT assay as described^33,34^. In brief, 50 μl of a 0.5 mg/ml solution of MTT (3-(4,5-dimethyl-2-thiazolyl)-2,5-diphenyl-2H-tetrazolium bromide) (Sigma, USA) was added to the washed biofilms for 1 h at 37 °C, followed by addition of 150 μl of PBS. After discarding the supernatant, the tetrazolium formed within the biofilms was dissolved in 200 μl of dimethyl sulfoxide (DMSO) and quantified by reading the OD at 570 nm in the M200 Tecan microplate reader. Different dilutions of the test compounds in the absence of bacteria were used to measure background signals. The percentage metabolic activity in the biofilms was calculated by dividing the OD of treated samples on OD of control samples, multiplied with 100%.

### Cell cycle analysis

Cell cycle analysis was performed by a slight modification of the described protocols^58,59^. MDRSA CI-M was incubated in the absence or presence of 50 µg/ml AEA for 2 h, and then centrifuged at 2400×*g* for 5 min. The bacterial pellet was resuspended in 50 µl PBS, and then fixed by adding 1 ml methanol dropwise under constant vortex. After an overnight storage at − 20 °C, the samples were rehydrated in PBS, followed by a 30 min incubation with 1 µg/ml DAPI. After washing the bacteria in PBS, the fluorescence intensity of DAPI that reflects the DNA content of the cells, was measured by flow cytometry (Fortessa LSR Flow cytometer) with an excitation/emission of 355/450 nm.

### Nitrocefin assay

The β-lactamase activity was analyzed by using chromogenic cephalosporin derivate nitrocefin (APExBIO) as substrate^60^. As a result of hydrolysis, the absorbance shifts from intact yellow nitrocefin (380 nm) to degraded red nitrocefin (486 nm). A 10 mM stock solution of Nitrocefin was prepared in DMSO. An overnight culture of MDRSA CI-M was resuspended in PBS supplemented with 1% glucose to an OD_600nm_ of 2. The bacteria were then treated with different concentrations of AEA for 30 min and then nitrocefin was added to a final concentration of 0.1 mM. The absorbance at 486 nm was monitored in a kinetic study each minute for 60 min using the Tecan infinite M200 plate reader.

### Scanning electron microscopy

Untreated and AEA-treated MDRSA CI-M were fixed in 4% paraformaldehyde (Electron Microscopy Sciences) in DDW for 2 h and then washed in DDW and let dry. Thereafter, the samples were coated with iridium and visualized using a Magellan 400L High-Resolution Scanning Electron Microscope at 200×–10,000 × magnification. The lengths of the bacteria were measured using the Photoshop software. Images were captured randomly from 4–5 different areas at different magnifications. The lengths of the bacteria were measured using the Photoshop software. 65–70 bacteria were measured for each treatment group from 4–5 independent high magnification images.

### Transmission electron microscopy (TEM)

Untreated and AEA-treated MDRSA CI-M were fixed in 2% formaldehyde and 2.5% glutaraldehyde in 0.1 M sodium cacodylate buffer, pH 7.4 and then processed for TEM. Samples were cut and stained with uranyl acetate and lead citrate. The grids were viewed with Jeol JEM-1400 Plus TEM (Jeol, Tokyo, Japan) equipped with ORIUS SC600 CCD camera (Gatan, Abingdon, United Kingdom), and Gatan Microscopy Suite program (DigitalMicrograph, Gatan, UK). Images were captured randomly from 4–5 different areas at different magnifications. The percentage of bacteria with complete septum was calculated out of total number of bacteria recorder from 6 different images. 840–890 bacteria were counted for each treatment group.

### Real-time qPCR

MDRSA CI-M that have been incubated with 50 μg/ml of AEA for 4 h at 37 °C under constant shaking of 150 rpm, were centrifuged, and the pellet was resuspended in 1 ml of Tri-Reagent (Sigma) and transferred to 2 ml tubes containing 200 μl acid-washed glass beads (Sigma). After 3 disruptions for 45 s at a speed of 4.5 using a FastPrep cell disrupter (BIO101, Savant Instruments), and removal of the glass beads by centrifugation (2 min at 20,000×*g*), the RNA was purified by the standard Tri-Reagent protocol of Sigma. The purified RNA was reversed transcribed to cDNA using the qScript cDNA synthesis kit (Quantabio). Real-time qPCR was done in a CFX96 BioRad Connect Real-Time PCR apparatus using Power Sybr Green Master Mix (Applied Biosystems, Life Technologies) on 2 ng cDNA in the presence of 300 nM forward/reverse primer sets (Suppl. Table 1). PCR conditions included an initial heating at 50 °C for 2 min, an activation step at 95 °C for 10 min, followed by 40 cycles of amplification (95 °C for 15 s, 60 °C for 1 min). Calculations of fold-change in gene expression in the treated bacteria in comparison to control bacteria exposed to same concentration of ethanol (e.g., 0.5% ethanol for 50 µg/ml AEA) were done according to the 2^−∆∆Ct^ method^61^, where *gyrB*, *glyA*, *gmk*, *proC*, *recF*, *rho* and RNAII were used as internal standards^62^. These house-keeping genes were tested against each other and found to provide a fold value of 1. The fold-change of each of the tested genes was calculated against each of these 7 genes, and the average of the calculated fold-changes in the treated bacteria in comparison to control bacteria is presented.

### Membrane potential assay

The effect of AEA on the membrane potential of MDRSA CI-M was analyzed using the BacLight Membrane Potential Kit (Molecular Probes, Life Technologies) according to the manufacturer's instructions^63^. MDRSA was treated with various concentrations of AEA in TSBG for either 2 h at 37 °C or AEA was added immediately before analyzing the membrane potential. The bacteria were resuspended in PBS and 3,3′-diethyloxacarbocyanine iodide (DiOC_2_(3)) was added to a final concentration of 30 μM and after 30 min the bacteria were analyzed on flow cytometry (LSR-Fortessa flow cytometer, BD Biosciences) using the 488 nm excitation laser and collecting the data using the green (530 nm) and red (610/620 nm) filters. The BD FACSDiva software was used for the collection of data and the FCS Express 7 software for analyzing the data.

### Nile red membrane staining

Control or AEA-treated bacteria were exposed to 10 μg/ml nile red (APExBIO) for 30 min to stain the membranes^64^. After washing the cells in PBS, the bacteria were analyzed on flow cytometry (LSR-Fortessa instrument, BD Biosciences) using the 561 nm yellow-green laser excitation and collecting the data using the 635 nm filter.

### Membrane permeability assay

Control or AEA-treated bacteria were exposed to 10 μg/ml propidium iodide (PI) for 20 min. PI enters only bacteria with a damaged membrane due to its positive charge^65^. After washing the bacteria in PBS, the bacteria were analyzed on flow cytometry (LSR-Fortessa instrument) using the 488 nm laser excitation and collecting the data using the 610/620 nm filter.

### DAPI accumulation assay

Control or AEA-treated bacteria were exposed to 1 μg/ml DAPI for 30 min. After washing the bacteria in PBS, the bacteria were analyzed on flow cytometry (LSR-Fortessa instrument) using the 355 nm laser excitation and collecting the data using the 450 nm filter. DAPI is related to the efflux pump substrate Hoechst 33,342 that has been used by others in the accumulation assay^66^.

### Spinning disk microscopy

Control and AEA-treated bacteria were stained with 10 μg/ml nile red and 1 μg/ml DAPI for 30 min as described^64^. Thereafter, the bacteria were washed with PBS and fixed with 4% paraformaldehyde in PBS for 30 min. The fixed bacteria were washed with PBS and loaded on agarose pad to visualize them under a spinning disk microscope (Nikon Yokogawa W1 Spinning Disk with 50 μm pinholes). The Plan-Apochromat × 100 objective was used with the 561 nm excitation laser for nile red and 405 nm excitation laser for DAPI.

### Ethidium bromide (EtBr) efflux assays

The fluorescent compound EtBr is known to be pumped out of the bacteria through NorA and other efflux pumps^17^. We performed the EtBr efflux assay essential as described^67^. An overnight culture of MRSA in TBS was pelleted and resuspended in TSBG to an OD_600nm_ of 0.8 and loaded with EtBr by incubating the bacteria at room temperature for 20 min in the presence of 25 μM EtBr (Sigma) and 100 μM of the protonophore carbonyl cyanide 3-chlorophenylhydrazone (CCCP; Sigma). At the end of incubation, the bacteria were washed thrice in PBS and resuspended in Hank's balanced salt solution (HBSS) containing different concentrations of AEA and 200 μl aliquots were transferred to μClear black opaque 96 well microplates (Greiner Bio-One) and read in the M200 Tecan plate reader at excitation/emission wavelength of 485 nm/645 nm each 5 min for a period of 3 h. The samples were also analyzed by flow cytometry (LSR-Fortessa instrument) using the 488 nm excitation laser and the red 610/620 nm filter. The readings were done each 15 min for 120 min.

### Measurements of intracellular Norfloxacin levels

MRSA CI-M was incubated with 25 μg/ml Norfloxacin in the absence or presence of 25 μg/ml AEA for 2 h at 37 °C in TSBG. Bacteria grown in the absence or presence of AEA alone served as unstained controls. At the end of incubation, the bacteria were washed and resuspended in TSBG with or without 25 μg/ml AEA and incubated at 37 °C for 3 h. Then the bacteria were washed, resuspended in PBS, and the intracellular norfloxacin level measured by flow cytometry (Fortessa LSR Flow cytometer) with an excitation/emission of 355/450 nm.

### Cell wall synthesis assay

Cell wall synthesis assay was performed according to the method described by Sugimoto et al.^64^. MDRSA CI-M was incubated in the presence or absence of 50 µg/ml AEA and/or 50 µg/ml methicillin for 2 h in TSBG at 37 °C. Then the samples were splitted into two. One series got 10 µM Sortase substrate I, FRET (Anaspec, CA, USA), while the other remained unstained. After a 2.5 h incubation at 37 °C, the bacteria were washed and the fluorescence intensity of the samples were read in the Tecan M200 plate reader with an excitation/emission of 340/490 nm.

### Western blot analysis of PBP2a protein level

Extraction of membrane proteins from crude bacterial membrane preparations was performed essential as described^68^. An equal amount (OD = 4) of untreated and 2 h AEA-treated MDRSA CI-M were washed with PBS containing 1 mM phenylmethylsulfonyl fluoride (PMSF; Sigma) and 1% complete protease inhibitors (PIC, Roche), and then incubated in 50 µl buffer K (50 mM Triethanolamine (TAE) (BioPLUS chemicals, bioWORLD), 250 mM sucrose, 1 mM EDTA, pH 7.5) supplemented with 1% PIC, 1 mM PMSF, 50 µg/ml lysostaphin (ProSpec-Tany TechnoGene Ltd, Israel), 10 µg/ml DNase (Sigma), 1 mM MgSO_4_, for 10 min at 37 °C. At the end of incubation, 350 µl of 750 mM aminocaproic acid (Sigma) was added. After three freeze–thaw cycles in liquid nitrogen 1 min/20 °C water 1 min, n-dodecyl β-D-maltoside (Sigma) was added to a final concentration of 1.25% and the samples incubated 1 h on ice with occasional mixing to allow the extraction of membrane proteins. Then the samples were centrifuged for 20 min at 20,000xg at 4 °C to remove insolubilized material. Nine volumes of the supernatant was mixed with one volume of 10 × blue native loading buffer (5% Coomassie brilliant blue G-250 (Merck), 250 mM aminocaproic acid and 25% glycerol), and 10 µl was run on a 3–12% gradient native blue PAGE as described by Zilkenat et al.^68^ until the front blue band went out of the gel. The proteins were transferred to a 0.22 μm PVDF Plus membrane, and immunoblotted with rabbit anti-PBP2a primary antibody (RayBiotech Inc., USA) followed by HRP-conjugated goat-anti-rabbit IgG (Jackson ImmunoResearch Laboratories Inc., USA) and enhanced chemiluminescence reaction (1.25 mM Luminol, 200 µM p-Cumaric acid, 3 mM H_2_O_2_) that was detected using the BioRad ChemiDoc Imager. The relative intensity of the bands was calculated by the densitometry using the Photoshop software. Gel loading control was done by staining the gel with Pierce silver stain kit (Thermo Scientific, IL, USA).

### Statistical analysis

The experiments were performed in experimental triplicates and repeated twice. The data are expressed as the average ± standard error. Statistical analysis was performed using the Microsoft excel software. Student's t-test was used to compare control and treated samples, with a p value less than 0.05 considered significant.

## Supplementary Information

Supplementary Information 1.

Supplementary Information 2.

## References

[1] Guo Y, Song G, Sun M, Wang J, Wang Y (2020). Prevalence and therapies of antibiotic-resistance in *Staphylococcus aureus*. Front. Cell Infect. Microbiol..

[2] Turner NA (2019). Methicillin-resistant *Staphylococcus aureus*: An overview of basic and clinical research. Nat. Rev. Microbiol..

[3] Lakhundi S, Zhang K (2018). Methicillin-resistant *Staphylococcus aureus*: Molecular characterization, evolution, and epidemiology. Clin. Microbiol. Rev..

[4] Karaman R, Jubeh B, Breijyeh Z (2020). Resistance of Gram-positive bacteria to current antibacterial agents and overcoming approaches. Molecules.

[5] Peacock SJ, Paterson GK (2015). Mechanisms of methicillin resistance in *Staphylococcus aureus*. Annu. Rev. Biochem..

[6] Blair JM, Webber MA, Baylay AJ, Ogbolu DO, Piddock LJ (2015). Molecular mechanisms of antibiotic resistance. Nat. Rev. Microbiol..

[7] Hall CW, Mah TF (2017). Molecular mechanisms of biofilm-based antibiotic resistance and tolerance in pathogenic bacteria. FEMS Microbiol. Rev..

[8] Li X-Z, Mayers DL (2017). Antimicrobial Drug Resistance: Mechanisms of Drug Resistance.

[9] Pinho MG, de Lencastre H, Tomasz A (2001). An acquired and a native penicillin-binding protein cooperate in building the cell wall of drug-resistant *staphylococci*. Proc. Natl. Acad. Sci. U.S.A..

[10] Evans J, Hannoodee M, Wittler M (2020). StatPearls (StatPearls Publishing Copyright © 2020.

[11] Chan LC (2015). Ceftobiprole- and ceftaroline-resistant methicillin-resistant *Staphylococcus aureus*. Antimicrob. Agents Chemother..

[12] Lozano C (2020). Human mecC-carrying MRSA: Clinical implications and risk factors. Microorganisms.

[13] Hooper DC (2000). Mechanisms of action and resistance of older and newer fluoroquinolones. Clin. Infect. Dis..

[14] Papkou A, Hedge J, Kapel N, Young B, MacLean RC (2020). Efflux pump activity potentiates the evolution of antibiotic resistance across *S. aureus* isolates. Nat. Commun..

[15] Costa SS, Viveiros M, Amaral L, Couto I (2013). Multidrug efflux pumps in *Staphylococcus aureus*: An update. Open Microbiol. J..

[16] Lekshmi M (2018). Modulation of antimicrobial efflux pumps of the major facilitator superfamily in *Staphylococcus aureus*. AIMS Microbiol..

[17] Patel D, Kosmidis C, Seo SM, Kaatz GW (2010). Ethidium bromide MIC screening for enhanced efflux pump gene expression or efflux activity in *Staphylococcus aureus*. Antimicrob. Agents Chemother..

[18] Blair JMA, Piddock LJV (2016). How to measure export via bacterial multidrug resistance efflux pumps. MBio.

[19] Whittle EE (2019). Flow cytometric analysis of efflux by dye accumulation. Front. Microbiol..

[20] Salaheen S, Peng M, Joo J, Teramoto H, Biswas D (2017). Eradication and sensitization of methicillin resistant *Staphylococcus aureus* to methicillin with bioactive extracts of berry pomace. Front. Microbiol..

[21] Thai KM (2015). Virtual screening for novel *Staphylococcus aureus* NorA efflux pump inhibitors from natural products. Med. Chem..

[22] García-Fernández E (2017). Membrane microdomain disassembly inhibits MRSA antibiotic resistance. Cell.

[23] Baugh S, Phillips CR, Ekanayaka AS, Piddock LJ, Webber MA (2014). Inhibition of multidrug efflux as a strategy to prevent biofilm formation. J. Antimicrob. Chemother..

[24] Kvist M, Hancock V, Klemm P (2008). Inactivation of efflux pumps abolishes bacterial biofilm formation. Appl. Environ. Microbiol..

[25] Alav I, Sutton JM, Rahman KM (2018). Role of bacterial efflux pumps in biofilm formation. J. Antimicrob. Chemother..

[26] Yoshikai H (2016). Multidrug-resistance transporter AbcA secretes *Staphylococcus aureus* cytolytic toxins. J. Infect. Dis..

[27] Zaman M, Andreasen M (2020). Cross-talk between individual phenol-soluble modulins in *Staphylococcus aureus* biofilm enables rapid and efficient amyloid formation. Elife.

[28] Truong-Bolduc QC, Dunman PM, Strahilevitz J, Projan SJ, Hooper DC (2005). MgrA is a multiple regulator of two new efflux pumps in *Staphylococcus aureus*. J. Bacteriol..

[29] He X, Ahn J (2011). Differential gene expression in planktonic and biofilm cells of multiple antibiotic-resistant *Salmonella Typhimurium* and *Staphylococcus aureus*. FEMS Microbiol. Lett..

[30] Trotonda MP, Tamber S, Memmi G, Cheung AL (2008). MgrA represses biofilm formation in *Staphylococcus aureus*. Infect. Immun..

[31] Zimmermann S (2019). Clinically approved drugs inhibit the *Staphylococcus aureus* multidrug NorA efflux pump and reduce biofilm formation. Front. Microbiol..

[32] Abd El-Baky RM, Sandle T, John J, Abuo-Rahma GEA, Hetta HF (2019). A novel mechanism of action of ketoconazole: Inhibition of the NorA efflux pump system and biofilm formation in multidrug-resistant *Staphylococcus aureus*. Infect. Drug Resist..

[33] Feldman M, Smoum R, Mechoulam R, Steinberg D (2018). Antimicrobial potential of endocannabinoid and endocannabinoid-like compounds against methicillin-resistant *Staphylococcus aureus*. Sci. Rep..

[34] Feldman M, Smoum R, Mechoulam R, Steinberg D (2020). Potential combinations of endocannabinoid/endocannabinoid-like compounds and antibiotics against methicillin-resistant *Staphylococcus aureus*. PLoS ONE.

[35] Sionov RV, Feldman M, Smoum R, Mechoulam R, Steinberg D (2020). Anandamide prevents the adhesion of filamentous *Candida albicans* to cervical epithelial cells. Sci. Rep..

[36] Lu HC, Mackie K (2016). An introduction to the endogenous cannabinoid system. Biol. Psychiatry.

[37] Ohno-Shosaku T, Kano M (2014). Endocannabinoid-mediated retrograde modulation of synaptic transmission. Curr. Opin. Neurobiol..

[38] Mechoulam R, Parker LA (2013). The endocannabinoid system and the brain. Annu. Rev. Psychol..

[39] Pacher P, Kogan NM, Mechoulam R (2020). Beyond THC and endocannabinoids. Annu. Rev. Pharmacol. Toxicol..

[40] Bedse G (2017). Functional redundancy between canonical endocannabinoid signaling systems in the modulation of anxiety. Biol. Psychiatry.

[41] Gobbi G (2005). Antidepressant-like activity and modulation of brain monoaminergic transmission by blockade of anandamide hydrolysis. Proc. Natl. Acad. Sci. U.S.A..

[42] Acharya N (2017). Endocannabinoid system acts as a regulator of immune homeostasis in the gut. Proc. Natl. Acad. Sci. U.S.A..

[43] Jackson AR, Nagarkatti P, Nagarkatti M (2014). Anandamide attenuates Th-17 cell-mediated delayed-type hypersensitivity response by triggering IL-10 production and consequent microRNA induction. PLoS ONE.

[44] Chiurchiù V (2016). Anandamide suppresses proinflammatory T cell responses in vitro through type-1 Cannabinoid receptor-mediated mTOR inhibition in human keratinocytes. J. Immunol..

[45] Engel MA (2008). Ulcerative colitis in AKR mice is attenuated by intraperitoneally administered anandamide. J. Physiol. Pharmacol..

[46] Magiorakos AP (2012). Multidrug-resistant, extensively drug-resistant and pandrug-resistant bacteria: An international expert proposal for interim standard definitions for acquired resistance. Clin. Microbiol. Infect..

[47] Weinstein ZB (2018). Modeling the impact of drug interactions on therapeutic selectivity. Nat. Commun..

[48] Monteiro JM (2015). Cell shape dynamics during the staphylococcal cell cycle. Nat. Commun..

[49] Bottomley AL (2014). *Staphylococcus aureus* DivIB is a peptidoglycan-binding protein that is required for a morphological checkpoint in cell division. Mol. Microbiol..

[50] Willing S, Dyer E, Schneewind O, Missiakas D (2020). FmhA and FmhC of *Staphylococcus aureus* incorporate serine residues into peptidoglycan crossbridges. J. Biol. Chem..

[51] McCarthy H (2015). Methicillin resistance and the biofilm phenotype in *Staphylococcus aureus*. Front. Cell Infect. Microbiol..

[52] Di Scala C, Fantini J, Yahi N, Barrantes FJ, Chahinian H (2018). Anandamide revisited: How cholesterol and ceramides control receptor-dependent and receptor-independent signal transmission pathways of a lipid neurotransmitter. Biomolecules.

[53] Reens AL (2018). A cell-based infection assay identifies efflux pump modulators that reduce bacterial intracellular load. PLoS Pathog..

[54] Devane WA (1992). Isolation and structure of a brain constituent that binds to the cannabinoid receptor. Science.

[55] White RL, Burgess DS, Manduru M, Bosso JA (1996). Comparison of three different in vitro methods of detecting synergy: Time-kill, checkerboard, and E test. Antimicrob. Agents Chemother..

[56] Bauer AW, Kirby WM, Sherris JC, Turck M (1966). Antibiotic susceptibility testing by a standardized single disk method. Am. J. Clin. Pathol..

[57] Farkash Y, Feldman M, Ginsburg I, Steinberg D, Shalish M (2019). Polyphenols inhibit *Candida albicans* and *Streptococcus mutans* biofilm formation. Dent. J. (Basel).

[58] Sionov RV, Netzer E, Shaulian E (2013). Differential regulation of FBXW7 isoforms by various stress stimuli. Cell Cycle.

[59] Button DK, Robertson BR (2001). Determination of DNA content of aquatic bacteria by flow cytometry. Appl. Environ. Microbiol..

[60] Hugonnet JE, Blanchard JS (2007). Irreversible inhibition of the *Mycobacterium tuberculosis* beta-lactamase by clavulanate. Biochemistry.

[61] Livak KJ, Schmittgen TD (2001). Analysis of relative gene expression data using real-time quantitative PCR and the 2(-Delta Delta C(T)) method. Methods.

[62] Theis T, Skurray RA, Brown MH (2007). Identification of suitable internal controls to study expression of a *Staphylococcus aureus* multidrug resistance system by quantitative real-time PCR. J. Microbiol. Methods.

[63] Shapiro HM (2000). Membrane potential estimation by flow cytometry. Methods.

[64] Sugimoto A, Maeda A, Itto K, Arimoto H (2017). Deciphering the mode of action of cell wall-inhibiting antibiotics using metabolic labeling of growing peptidoglycan in *Streptococcus pyogenes*. Sci. Rep..

[65] Boulos L, Prévost M, Barbeau B, Coallier J, Desjardins R (1999). LIVE/DEAD BacLight : Application of a new rapid staining method for direct enumeration of viable and total bacteria in drinking water. J. Microbiol. Methods.

[66] Richmond GE, Chua KL, Piddock LJ (2013). Efflux in *Acinetobacter baumannii* can be determined by measuring accumulation of H33342 (bis-benzamide). J. Antimicrob. Chemother..

[67] Felicetti T (2018). 2-Phenylquinoline *S. aureus* NorA efflux pump inhibitors: Evaluation of the importance of methoxy group introduction. J. Med. Chem..

[68] Zilkenat S (2017). Blue Native PAGE analysis of bacterial secretion complexes. Methods Mol. Biol..

