# Supplementary material for: Anandamide alters the membrane properties, halts the cell division and prevents drug efflux in multidrug resistant Staphylococcus aureus
Source: Sci Rep. 2021 Apr 22;11:8690. doi: 10.1038/s41598-021-88099-6 (PMC8062478; doi:10.1038/s41598-021-88099-6)
Supplement: Supplementary file 1 — Supplementary Information 1. [file 41598_2021_88099_MOESM1_ESM.pdf]

Supplementary Data

ANANDAMIDE ALTERS THE MEMBRANE PROPERTIES, HALTS THE CELL DIVISION AND PREVENTS DRUG EFFLUX IN MULTIDRUG RESISTANT *STAPHYLOCOCCUS AUREUS*

Shreya Banerjee, Ronit Vogt Sionov, Mark Feldman, Reem Smoum, Raphael Mechoulam, and Doron Steinberg

Control 2h

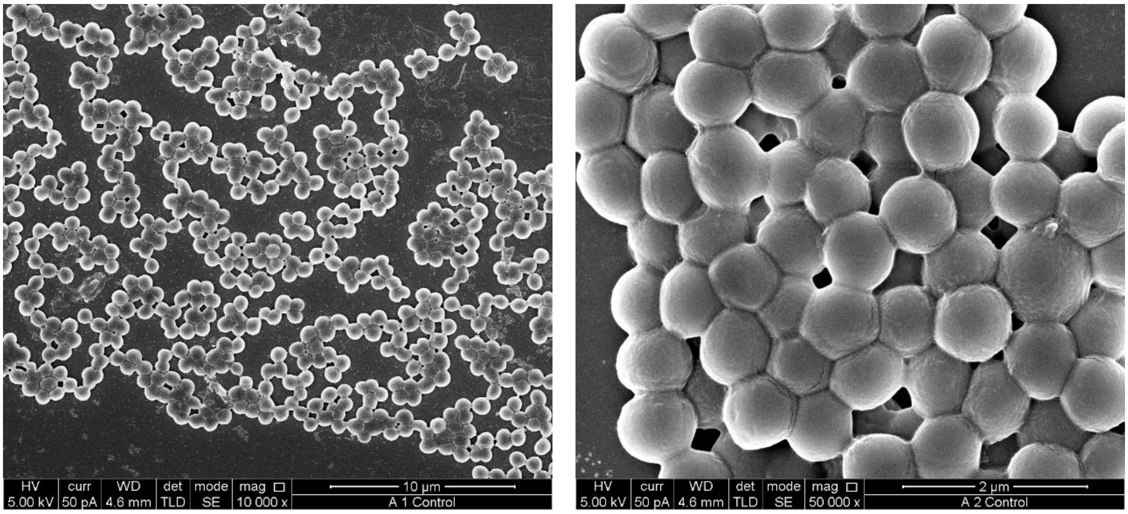

25 µg/ml AEA 2h

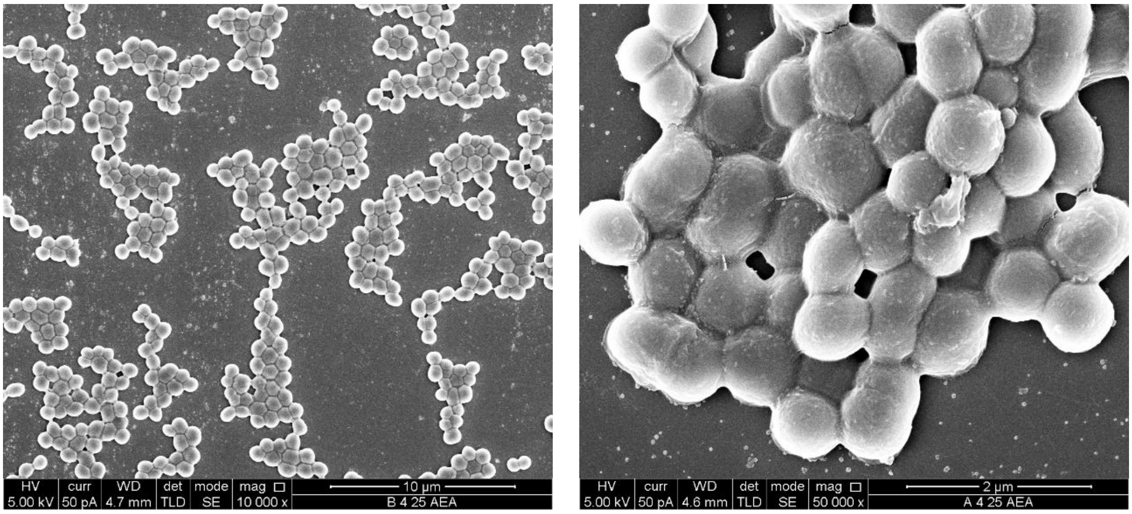

Suppl. Figure 1. SEM pictures of control MDRSA CI-M bacteria incubated for 2h or bacteria treated with 25 µg/ml AEA for 2h. Two different magnifications are shown.

Control 2h

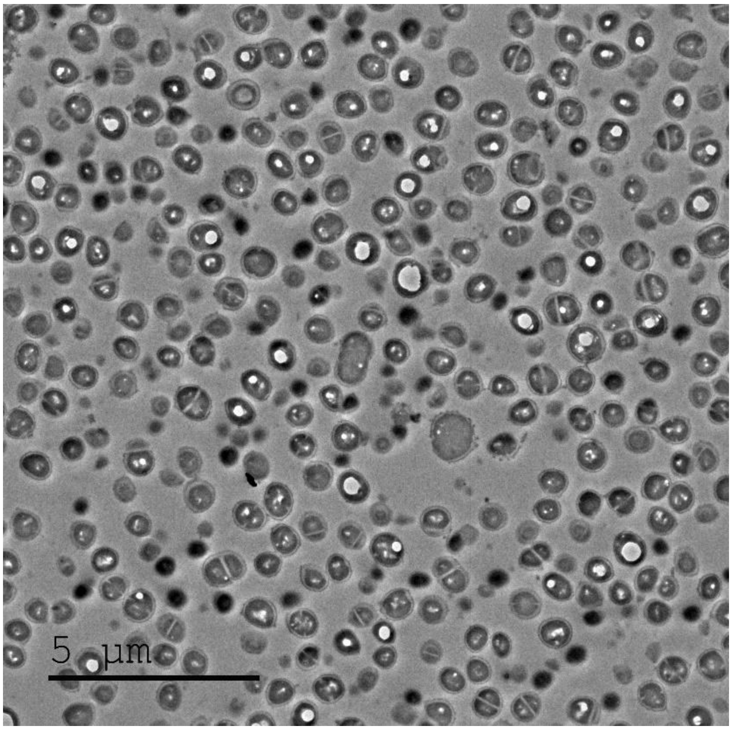

50 µg/ml AEA 2h

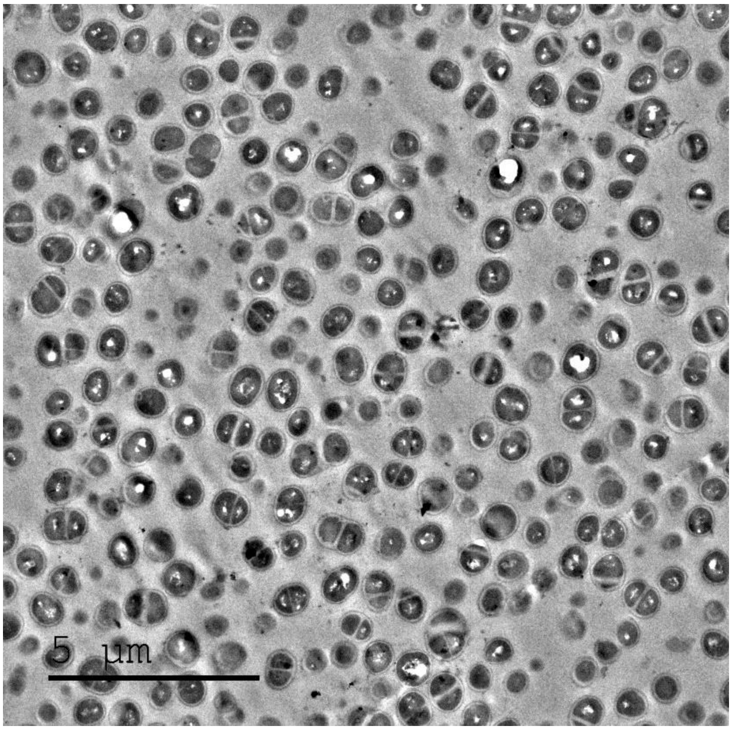

Suppl. Figure 2. TEM pictures of control MDRSA CI-M bacteria incubated for 2h or bacteria treated with 50 µg/ml AEA for 2 h. A low magnification is shown to get a general overview of the two cultures.

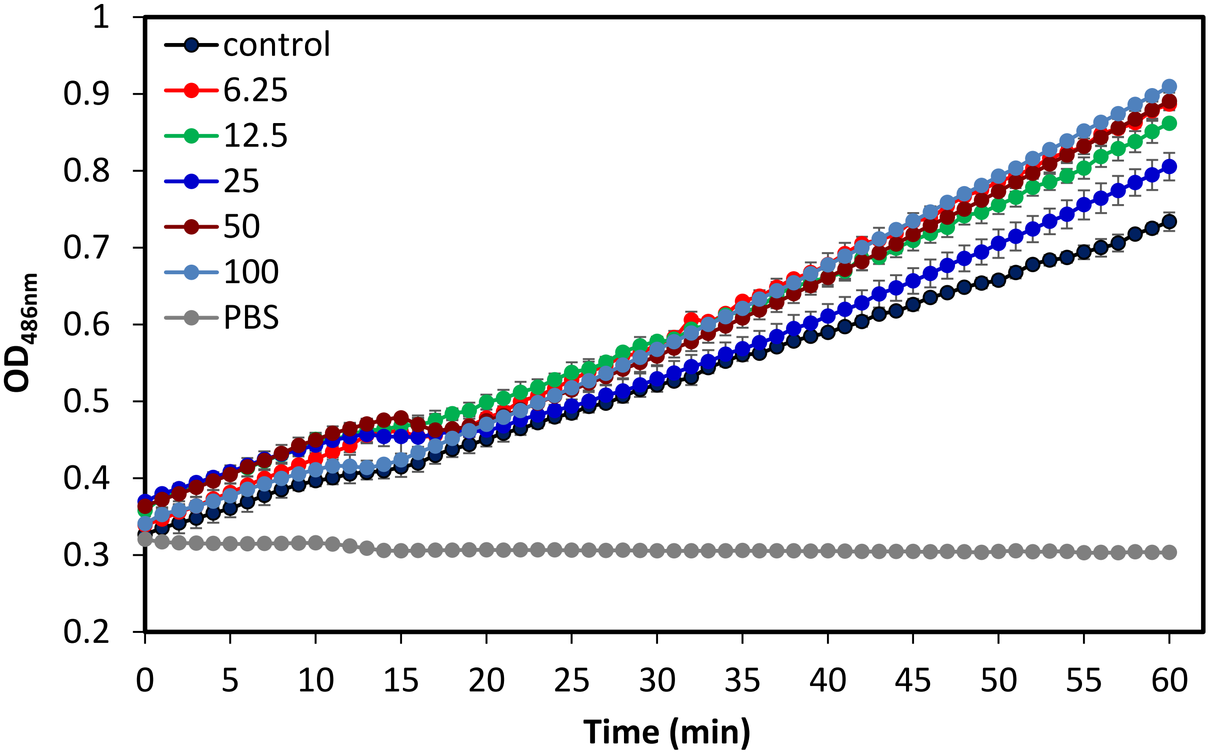

Suppl. Fig. 3. Anandamide (AEA) does not affect the β-lactamase activity. Control and AEA-treated MDRSA CI-M after a 30 min incubation were exposed to 0.1 mM Nitrocefin, and the hydrolysis of nitrocefin was monitored each minute for 60 min by measuring the degraded red nitrocefin that absorbs at 486nm.

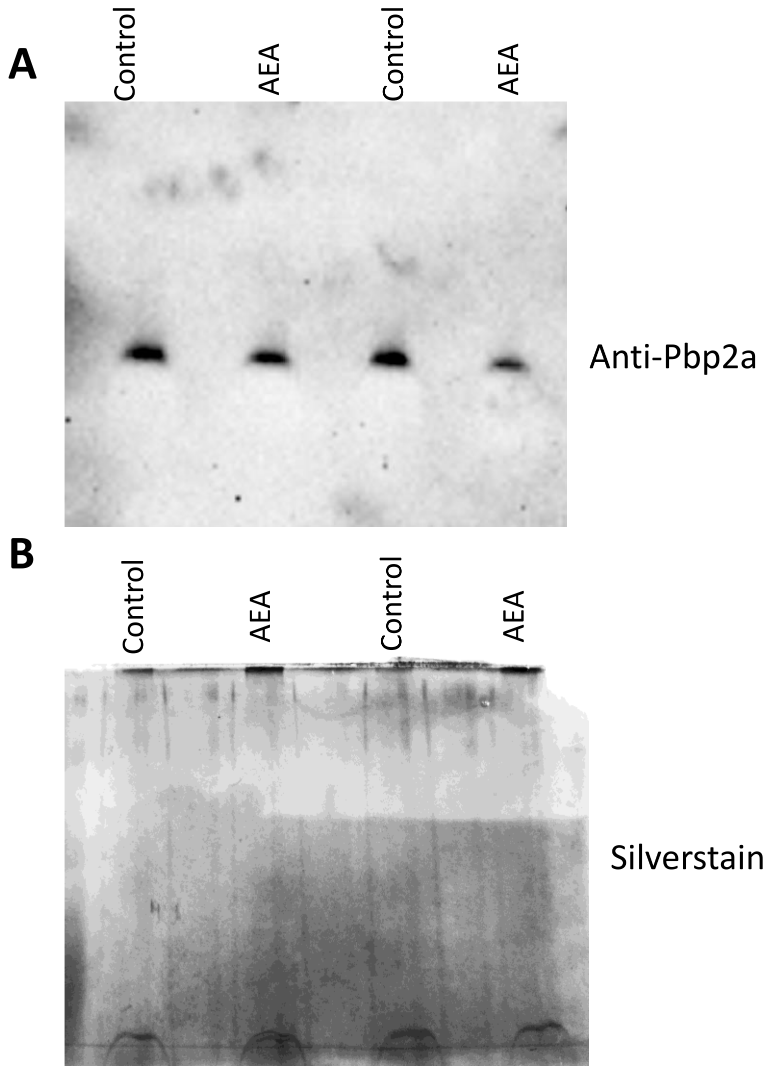

Suppl. Fig. 4. A. The original Western blot of Pbp2a of Fig. 11B run in a non-reduced 3-12% gradient PAGE. **B.** The Silverstain of the same samples in the PAGE gel.
