# Supplementary material for: Anandamide alters the membrane properties, halts the cell division and prevents drug efflux in multidrug resistant Staphylococcus aureus
Source: Sci Rep. 2021 Apr 22;11:8690. doi: 10.1038/s41598-021-88099-6 (PMC8062478; doi:10.1038/s41598-021-88099-6)
Supplement: Supplementary file 2 — Supplementary Information 2. [file 41598_2021_88099_MOESM2_ESM.pdf]

Anandamide alters the membrane properties, halts the cell division and prevents drug efflux in multidrug resistant *Staphylococcus aureus*

Shreya Banerjee, Ronit Vogt Sionov, Mark Feldman, Reem Smoum,
Raphael Mechoulam, and Doron Steinberg

Suppl. Table 1. Primers used for real-time PCR of *S. aureus*

Gene	Forward Primer	Reverse Primer	Ref
16S rRNA	CCAGCAGCCGCGGTAAT	CGCGCTTTACGCCCAATA	1
agrA	AAAGTTGCAGCGATGGATTT	ATGGGCAATGAGTCTGTGAG	1
agrC	CATTCGCGTTGCATTTATTG	CCTAAACCACGACCTTCACC	2
atl	GACCCTGCTATTGTCCAACC	CGCTGATTGATTAGCACGAG	3
aur	CAAAAGAGTGATGCGGTCAA	AGGTGCATGAACACCATCAA	2
capA	CAGTTAAAGTCGCACCAA	GAACCCAATACAGGCAAT	1
clfA	ACCCAGGTTCAGATTCTGGCAGCG	TCGCTGAGTCGGAATCGCTTGCT	1
cna	AATAGAGGCGCCACGACCGT	GTGCCTTCCCAAACCTTTTGAGCA	1
crtM	ATCCAGAACCACCCGTTTTT	GCGATGAAGGTATTGGCATT	2
crtN	GATGAAGCTTTGACGCAACA	TTCGCATGATACGTTTGCTC	2
divIB	CGACTCGATTGATGAGGAAAC	TTGTCGCTTACGTCTTAACTTCC	3
divIC	ATGCGTGTTGTTCGTAGGC	CGCTCCTGTGCATCAATATC	3
divIVA	GATCGCGTTTCCGTATGTTAG	TCAAGCGTCACTTGTTCAGC	3
ebps	GGTGCAGCTGGTGCAATGGGTGT	GCTGCGCCTCCAGCCAAACCT	1
eno	TGCCGTAGGTGACGAAGGTGGTT	GCACCGTGTTCGCCTTCGAACT	1
ezrA	ATTTGGTGAGGCAGCAAGTC	GCTATATGGTTGTGCGCTTG	3
fib	CGTCAACAGCAGATGCGAGCG	TGCATCAGTTTTCGCTGCTGGTTT	1
fmhB	AAGCGAGGTACGACAGTAGAACG	CATCTCCATCTTCATGCAACGCA	4
fnbA	AAATTGGGAGCAGCATCAGT	GCAGCTGAATTCCCATTTTC	1
fnbB	AAGAAGCACCGAAAACTGTG	TCTCTGCAACTGCTGTAACG	2
ftsA	GGCGAGAAATTTCACAATGG	TGTGTCTTTGATTGCTTGTCG	3
ftsH	GATTGATGCTGTTGGTCGTC	CCGAAACCATCCATTTCAA	3
ftsL	GATACGCGAGGAAAGATTGC	ATCGTTCTCAAGGCTCATCC	3
ftzZ	AAAGCTGCAGAGGAATCTCG	TTTAGCAACGACTGGTGCTG	3
ftsW	CTTGTGTGTGTTGGGATTGC	TTGATGATCCACCAAAGCTG	3
glyA	CTACAAACTCACAGCCAC	GTATCGGAAGCGGTTATG	5
gmk	CCATCTGGAGTAGGTAAAGG	CTACGCCATCAACTTCAC	5
gtf	TGGTGACGCCGAAGGACTC	GCAGCACGAGCAGGAACAC	1
gyrA	TGGCCCAAGACTTTAGTTATCGTTATCC	TGGGGAGGAATATTTGTAGCCATACCTAC	6
gyrB	GGTGCTGGGCAAATACAAGT	TCCCACACTAAATGGTGCAA	2
hla	AATGAATCCTGTCGCTAATGCCGC	CTGAAGGCCAGGCTAAACCACTTT	1
hld	TAATTAAGGAAGGAGTGATTTCAATG	TTTTTAGTGAATTTGTTCACTGTGTC	1
icaA	ACACTTGCTGGCGCAGTCAA	TCTGGAACCAACATCCAACA	7
icaB	ATGGTCAAGCCCAGACAGAG	AGTATTTTCAATGTTTAAAGCA	7
icaC	CTTGGGTATTTGCACGCATT	GCAATATCATGCCGACACCT	1
kdpA	ATTGTTCGGTTTATTGTCC	CATCATACTGCCCATTTCT	1
lytA	TGGTGGTGCAAAGTTCATTC	TATCTGCCCAGCGAATGTC	3
lytM	TCAGCAAGTAAAGCGACAGC	TTTCAGGCATTGCATAGTCG	3
lytN	TGCCAATGACACCATTAGTAGAAC	ACCGTCGAAATCCCATCC	3
mecA	AACCGAAGATAAAAAAGAACC	GTCCGTAACCTGAATCAGC	1
mecR1	GTGCTCGTCTCCACGTTAATTCCA	GACTAACCGAAGAAGTCGTGTCAG	4
mdeA	CTTTCAGGTTACCTTGTTGAATATTTAAAC	ATCAATAGGTACTTTAATTGTAGTTCCAAC	1
mepA	ATGGTATAGGTTTCTTGTTTACTGGTATG	AATGATAATTGCACCTTGTAAAATGGC	1
mgrA	AGTACAATCTAACATACC	TTGCGATAAAGAAGAAGC	1
norA	GACATTTCACCAAGCCATCAA	TGCCATAAATCCACCAATCC	1

norB	GCTACACCATCAACAGATACAGCAA	ACTCAATGCGACGCCAAA	1
norC	TGGGTTGGAGATGGATTTTC	ACAATTAGCCCTGCAACGTC	1
nuc	GCGATTGATGGTGATACGGTT	AGCCAAGCCTTGACGAACTAAAGC	2
opp1C	CGTGCGTGTGATGTTATGTTGGCA	TTCGGCACCCATTCCAAACAATGC	4
oppF	GGTGGGCAACGTCAGCGTGTAA	GGACACTGCCTCGTCGCAAACA	4
pbp2	CGTTTCTACGAACATGGCGCAC	GGCACCTTCAGAACCAAATCCACCA	4
proC	GGCAGGTATTCCGATTGA	CCAGTAACAGAGTGTCCAAC	5
psmα	TATCAAAAGCTTAATCGAACAATTC	CCCCTTCAAATAAGATGTTCATATC	2
qacA/B	GGTGCTTTAATAATGCC	CCAGTCCAATCATGCCTGC	1
recF	AGTTATAGACACGGCACG	GCGTCGTCTTATTTGAGG	5
rho	GGAAGATACGACGTTCAGAC	GAAGCGGGTGGAAGTTTA	5
RNAII	TATGAATAAATGCGCTGATGATATACCACG	TTTTAAAGTTGATAGACCTAAACCACGACC	6
RNAIII	GCACTGAGTCCAAGGAAACTAACTCT	AGCCATCCCAACTTAATAACCATGT	2
rpoB	TCCTGTTGAACGCGCATGTAA	GCTGGTATGGCTCGTGATGGTA	8
saeR	AAGTGGCGACCATTACAT	CATTATTGCCTCAAATACGT	1
saeS	TGCCAATACCTTCATCGCTAA	CAATATCGAACGCCACTTGA	7
sarA	GACATACATCAGCGAAAA	TACGTTGTTGTGCATTAA	1
sdrM	CAACATGGCATTGGTTATTCTAC	ACAGCTGTTGGTTTAATAAAGC	1
sepA	GAAGTATGTACGATAACCTATTATATTATG GC	AAAGTCGCGCCTCTAAAATATGC	1
sepF	GCGGTACTGTTTATGCAATCG	TGGTCTGTAATGCTTCCAGCTAC	3
sle1	TCAGGATCTGCAACAACGAC	CCTTTACCAATTTCAGCACGAC	3
sspB	CCAGCAAATTGTTGTTGTGCTAG	AAGCCAAAGCCGATTCACACTC	7

References

1. Salaheen, S., Peng, M., Joo, J., Teramoto, H. & Biswas, D. Eradication and sensitization of methicillin resistant *Staphylococcus aureus* to methicillin with bioactive extracts of berry pomace. *Front Microbiol* **8**, 253, doi:10.3389/fmicb.2017.00253 (2017).
2. Valliammai, A. *et al.* 5-Dodecanolide interferes with biofilm formation and reduces the virulence of Methicillin-resistant Staphylococcus aureus (MRSA) through up regulation of agr system. *Sci Rep* **9**, 13744, doi:10.1038/s41598-019-50207-y (2019).
3. Wassmann, C. S., Højrup, P. & Klitgaard, J. K. Cannabidiol is an effective helper compound in combination with bacitracin to kill Gram-positive bacteria. *Sci Rep* **10**, 4112, doi:10.1038/s41598-020-60952-0 (2020).
4. Chung, P. Y., Chung, L. Y. & Navaratnam, P. Transcriptional profiles of the response of methicillin-resistant *Staphylococcus aureus* to pentacyclic triterpenoids. *PLoS One* **8**, e56687, doi:10.1371/journal.pone.0056687 (2013).
5. Theis, T., Skurray, R. A. & Brown, M. H. Identification of suitable internal controls to study expression of a *Staphylococcus aureus* multidrug resistance system by quantitative real-time PCR. *J Microbiol Methods* **70**, 355-362, doi:10.1016/j.mimet.2007.05.011 (2007).
6. Eleaume, H. & Jabbouri, S. Comparison of two standardisation methods in real-time quantitative RT-PCR to follow *Staphylococcus aureus* genes expression during *in vitro* growth. *J Microbiol Methods* **59**, 363-370, doi:10.1016/j.mimet.2004.07.015 (2004).
7. Sethupathy, S., Vigneshwari, L., Valliammai, A., Balamurugan, K. & Pandian, S. K. L-Ascorbyl 2, 6-Dipalmitate inhibits biofilm and virulence in methicillin-resistant *Staphylococcus aureus* and prevents triacylglyceride accumulation in *Caenorhabditis elegans*. *RSC Adv* **7**, 23392-23406 doi:10.1039/c7ra02934a (2017).
8. Baroja, M. L. *et al.* The SaeRS Two-Component System Is a Direct and Dominant Transcriptional Activator of Toxic Shock Syndrome Toxin 1 in *Staphylococcus aureus*. *J Bacteriol* **198**, 2732-2742, doi:10.1128/jb.00425-16 (2016).
